# Piezo1 promotes EMT and tumor progression in HNSCC through a YAP1-Associated Galectin-1 axis

**DOI:** 10.3389/fcell.2026.1907708

**Published:** 2026-09-10

**Authors:** Fenqian Yuan, Jingkang Yong, Xueming Liu, Yifeng Wang, Dan Zhao

**Affiliations:** 1 Department of Head and Neck Surgery, Jiangxi Cancer Hospital & Institute, Jiangxi Clinical Research Center for Cancer, The Second Affiliated Hospital of Nanchang Medical College, Jiangxi Key Laboratory of Oncology, Nanchang, Jiangxi, China; 2 Department of Anesthesiology, Jiangxi Cancer Hospital & Institute, Jiangxi Clinical Research Center for Cancer, The Second Affiliated Hospital of Nanchang Medical College, Jiangxi Key Laboratory of Oncology, Nanchang, Jiangxi, China

**Keywords:** EMT, epithelial to mesenchymal transformation, galectin-1, HNSCC, Piezo1, Yap1

## Abstract

**Background:**

Head and neck squamous cell carcinoma (HNSCC) is an aggressive malignancy in which epithelial–mesenchymal transition (EMT) contributes to tumor progression. Piezo1 and Galectin-1 have each been implicated in cancer progression, but their functional relationship in HNSCC remains incompletely understood. We investigated Piezo1-associated mechanosensitive signaling and its potential relationship with Galectin-1 in HNSCC.

**Methods:**

Public transcriptomic and proteomic analyses were integrated with validation using 16 paired oral squamous cell carcinoma (OSCC) tumor and adjacent non-tumor specimens. Functional studies were performed in HNSCC cell lines using genetic and pharmacological modulation of PIEZO1/Piezo1 and Galectin-1-associated signaling. Two independent SCC-9 xenograft studies were conducted in separate cohorts (n = 6 per group): one comparing vehicle, Yoda1, and GsMTx4, and the other comparing vehicle, GsMTx4, OTX008, and their combination.

**Results:**

Piezo1 was consistently upregulated in HNSCC datasets, OSCC tissues, and HNSCC cell lines, whereas its association with survival was modest and not uniformly reproduced across analytical platforms. Piezo1 activation was associated with increased Ca^2+^-associated signaling, nuclear YAP1 accumulation, and EMT-related phenotypes, whereas PIEZO1 silencing and pharmacological inhibition of mechanosensitive channel activity attenuated these effects. Galectin-1 emerged as a Piezo1-responsive candidate effector associated with YAP1 signaling and EMT-related phenotypes. OTX008 treatment reduced proliferation, wound closure, invasion, and EMT-related marker changes. In xenografts, combined GsMTx4 and OTX008 treatment produced greater reductions in tumor growth, Ki67 positivity, endpoint CD31 fluorescence intensity, and EMT-associated marker changes than either monotherapy at the tested doses.

**Conclusion:**

These findings support an important contribution of Piezo1-associated mechanosensitive signaling to EMT-related phenotypes and tumor progression in HNSCC and support Galectin-1 as a functionally relevant, Piezo1-responsive candidate effector associated with YAP1 signaling. The greater antitumor effects observed with combined GsMTx4 and OTX008 treatment support further investigation of co-targeting mechanosensitive channel activity and Galectin-1 in HNSCC.

## 1 Introduction

Head and neck cancers are the sixth most common malignancies worldwide, with head and neck squamous cell carcinoma (HNSCC) representing the predominant subtype. HNSCC is characterized by a relatively high incidence, and most patients present with locally advanced disease or distant metastases at diagnosis. Despite advances in surgery, radiotherapy, and chemotherapy, the 5-year survival rate for HNSCC remains moderate (Barsouk et al., 2023; Du et al., 2019). Local recurrence and distant metastasis remain the leading causes of poor clinical outcomes.

Epithelial–mesenchymal transition (EMT) is recognized as a central biological process in the initiation and progression of HNSCC (Baumeister et al., 2021). During EMT, tumor cells lose their epithelial characteristics—such as polarity and strong cell–cell adhesion—and acquire mesenchymal traits that promote migration and invasion. In HNSCC, EMT not only drives invasion and metastasis but also contributes to therapeutic resistance, immune evasion, and stem-like properties (Thierauf et al., 2017). Several signaling pathways, including TGF-β, Wnt/β-catenin, PI3K/AKT, and Hippo-YAP signaling, regulate EMT through transcription factors such as Snail, Slug, Twist, and the ZEB family, thereby accelerating malignant progression (González-González et al., 2021; Graves et al., 2014). Understanding the molecular regulation of EMT in HNSCC is therefore essential for identifying novel therapeutic targets and improving patient outcomes.

Piezo1, a mechanosensitive ion channel discovered by Coste et al., in 2010, is activated by mechanical cues such as shear stress, stretch, and matrix stiffness (Coste et al., 2010). Upon activation, Piezo1 mediates calcium influx and regulates processes including angiogenesis, erythrocyte homeostasis, and immune responses. Recent studies show that Piezo1 is highly expressed in several solid tumors—including breast, lung, pancreatic, and oral squamous cell carcinoma—where its expression correlates with invasion, metastasis, and poor prognosis (Jiang et al., 2022; Peng et al., 2025). By triggering calcium-dependent pathways such as MAPK/ERK, AKT/mTOR, and Hippo-YAP signaling, Piezo1 promotes proliferation, migration, and EMT, thus driving tumor progression (Deng et al., 2025; Xiong et al., 2022). In oral squamous cell carcinoma (OSCC), Piezo1 is a transcriptional target of the YAP pathway and is co-expressed with the proliferation marker Ki67, suggesting a potential role in tumor growth (Hasegawa et al., 2021). However, its precise function in HNSCC and its connection to EMT remain unclear.

Galectin-1 (Gal-1), encoded by *LGALS1*, is a β-galactoside-binding protein of the galectin family that acts as a context-dependent regulator of EMT through extensive crosstalk with multiple signaling pathways. It has been implicated in diverse biological processes, including tumor progression (Perez-Moreno et al., 2024). In OSCC, Gal-1 expression increases with tumor grade and stage and is strongly linked to invasion, metastasis, and formation of an immunosuppressive microenvironment (Salunkhe et al., 2020). In HNSCC, Gal-1 drives pre-metastatic niche formation and distant metastasis through STING-dependent inflammatory signaling and myeloid-derived suppressor cell-mediated immunosuppression (Nambiar et al., 2023). This makes Gal-1 a key contributor to resistance against immunotherapy (Nambiar et al., 2019). Inhibition of Gal-1 has been shown to reprogram the tumor microenvironment, converting “cold tumors” into “hot tumors” and enhancing responsiveness to immunotherapy. The Gal-1 inhibitor OTX008 has demonstrated antitumor effects in HNSCC by normalizing vasculature and suppressing tumor growth (Greer et al., 2022; Koonce et al., 2017). Yet, the specific relationship between Gal-1 and Piezo1 in HNSCC has not yet been fully elucidated.

In this study, we integrated public datasets, clinical samples, cellular experiments, and xenograft models to investigate Piezo1-associated signaling and its relationship with Galectin-1 in HNSCC. Our findings support an important contribution of Piezo1-associated signaling to EMT-related phenotypes and identify Galectin-1 as a functionally relevant, Piezo1-responsive candidate effector associated with YAP1 signaling. These results further suggest that combined targeting of mechanosensitive channel activity and Galectin-1 warrants investigation as a potential therapeutic strategy for HNSCC.

## 2 Materials and methods

### 2.1 Public database mining

Using “HNSCC” as the keyword, three Gene Expression Omnibus (GEO) datasets (GSE83519, GSE37991, and GSE30784) were retrieved and selected for expression analysis of target genes. Gene expression and survival correlations were further analyzed using the University of Alabama at Birmingham Cancer data analysis portal (UALCAN; accessed July–August 2025), based on The Cancer Genome Atlas (TCGA) and Clinical Proteomic Tumor Analysis Consortium (CPTAC) data. Kaplan–Meier survival curves and correlation analyses were generated using Gene Expression Profiling Interactive Analysis 2 (GEPIA2; accessed July–August 2025). Candidate genes potentially associated with *PIEZO1* were retrieved from Pathway Commons (v14), BioGRID (v4.4.248), STRING (v12.0), and IntAct (Release 250), all accessed in July–August 2025. Because no gene was shared across all four databases, the 15 genes common to Pathway Commons, BioGRID, and IntAct were retained and intersected with an HNSCC-related gene set retrieved from GeneCards (v5.25; accessed July–August 2025). GeneCards was used as a broad disease-relevance filter rather than as evidence of direct molecular interaction. Functional enrichment analysis of the seven overlapping genes was performed using the DAVID Knowledgebase (v2025_1; accessed July–August 2025) and was considered descriptive because of the limited gene-set size. *LGALS1* was prioritized for experimental validation based on its consistent upregulation across HNSCC datasets, increase during disease progression, positive correlation with *PIEZO1*, and established relevance to EMT.

### 2.2 Clinical sample collection

The collection and research use of paired human OSCC specimens were approved by the Institutional Ethics Committee of Jiangxi Cancer Hospital (approval no. 2025ky301). Written informed consent for the collection and research use of tissue specimens and associated clinical information was obtained from all participants before sample acquisition. Paired tumor and adjacent non-tumor tissues were obtained from the hospital biobank and stored at −80 °C. All procedures involving human specimens were conducted in accordance with the Declaration of Helsinki and applicable national and institutional requirements. Sixteen pathologically confirmed frozen tissue pairs were included in this study, comprising 11 stage II and 5 stage III cases. All samples were used for total protein extraction and subsequent Western blot analysis.

### 2.3 Cell culture

The following human cell lines were used: immortalized normal oral keratinocytes (OKF6/TERT1), dysplastic oral keratinocytes (DOK), pharyngeal squamous cell carcinoma cells (FaDu), metastatic HNSCC cells (Detroit 562), and tongue squamous cell carcinoma cells (SCC-9). OKF6/TERT1 cells were obtained through a custom procurement service provided by Fuheng Biology (Shanghai, China). Because these cells were acquired on a custom basis rather than as a routine catalogued product, no catalogue number was assigned. DOK cells were obtained from CancerTools (London, United Kingdom; Cat. No. 153251). FaDu, Detroit 562, and SCC-9 cells were purchased from Fuheng Biology (Cat. Nos. FH0297, FH0299, and FH1227, respectively).

OKF6/TERT1 cells were maintained in complete Keratinocyte Serum-Free Medium prepared using the Keratinocyte SFM kit, which included the basal medium and manufacturer-supplied recombinant human epidermal growth factor and bovine pituitary extract supplements (Gibco, Thermo Fisher Scientific; Cat. No. 17005042), with the addition of 1% penicillin–streptomycin (Servicebio, Wuhan, China; Cat. No. G4016). DOK cells were cultured in Dulbecco’s modified Eagle’s medium (DMEM; Servicebio; Cat. No. G4515) supplemented with 2 mM L-glutamine, 5 μg/mL hydrocortisone (Aladdin; Cat. No. H657409), 10% fetal bovine serum (Fuheng Biology; Cat. No. FH100-800), and 1% penicillin–streptomycin. FaDu and Detroit 562 cells were cultured in minimum essential medium (MEM; Servicebio; Cat. No. G4552) supplemented with 10% fetal bovine serum and 1% penicillin–streptomycin. SCC-9 cells were cultured in DMEM/Ham’s F-12 medium (Servicebio; Cat. No. G4612) supplemented with 10% fetal bovine serum and 1% penicillin–streptomycin. Unless otherwise specified, the same fetal bovine serum and penicillin–streptomycin products were used for all cell lines.

All cells were maintained at 37 °C in a humidified atmosphere containing 5% CO_2_. The identities of all cell lines were authenticated by short tandem repeat profiling, and all cultures tested negative for *mycoplasma* contamination before use. Cells were used within 20 passages after thawing, and parallel experiments were performed using cells of comparable passage numbers.

### 2.4 siRNA transfection

Small interfering RNAs (siRNAs) targeting *PIEZO1* were designed according to previously reported sequences (Lee et al., 2014; Lee et al., 2021; Savadipour et al., 2023). Three *PIEZO1*-targeting siRNAs with the sequences CAG​CGA​GAU​CUC​GCA​CUC​CAU​C, UAC​GAC​CUG​CUG​CAG​CUC​CUG, and ACC​CGC​UGG​CCA​UGC​AGU​UCU​U were mixed at an equimolar ratio and used as a pooled siRNA preparation. Cells were transfected with the siRNA pool by electroporation using a Neon Transfection System (Thermo Fisher Scientific, Waltham, MA, United States) according to the manufacturer’s instructions. A non-targeting siRNA was used as the negative control. *PIEZO1* knockdown efficiency was evaluated 72 h after transfection by Western blotting (Supplementary Figure S1A–C).

For *LGALS1* silencing, cells were transfected with an *LGALS1*-targeting siRNA (CCA​GCC​TGG​AAG​TGT​TGC​AGA) using the Neon Transfection System (Thermo Fisher Scientific, Waltham, MA, United States) according to the manufacturer’s instructions. A corresponding non-targeting siRNA was used as the negative control. Knockdown efficiency was assessed by Western blotting at 72 h after transfection (Supplementary Figure S1D-F). Unless otherwise specified, downstream functional assays were initiated after confirmation of gene silencing.

### 2.5 Reagents and pharmacological treatments

Yoda1, GsMTx4, OTX008, verteporfin, and recombinant human Galectin-1 were purchased from MedChemExpress (Monmouth Junction, NJ, United States). Stock solutions of Yoda1, GsMTx4, OTX008, and verteporfin were prepared in DMSO and diluted to the indicated working concentrations immediately before use. Recombinant human Galectin-1 was reconstituted in sterile PBS and diluted in culture medium to the indicated working concentration before use. Corresponding vehicle controls contained the same final concentration of solvent as the treatment groups. Drug concentrations and treatment durations for individual experiments are specified in the corresponding figure legends.

### 2.6 Cell proliferation assay

Cell proliferation was assessed using a cell counting assay. Cells were seeded in 6-well plates at appropriate densities, allowed to adhere, and cultured until logarithmic growth. Every 24 h, cells were harvested by 0.25% trypsin digestion and counted. Growth curves were plotted over 5 consecutive days. Drug treatments included Yoda1 (10 μM), GsMTx4 (1 and 10 μM for the proliferation assay and 10 μM for the remaining cellular assays) (Hope et al., 2019), and OTX008 (100 μM) (Greer et al., 2022).

### 2.7 Intracellular Calcium fluorescence measurement

Intracellular Ca^2+^-associated fluorescence was assessed using Cal-520 AM (Aladdin, Shanghai, China). Cells were seeded in culture plates and allowed to adhere before the indicated treatments. Cal-520 AM was prepared according to the manufacturer’s instructions and applied to cells at a final concentration of 5 μM. Cells were incubated with Cal-520 AM at 37 °C for 60 min in the dark, followed by washing with Hank’s balanced salt solution (HBSS) to remove excess extracellular dye. Cells were then allowed to equilibrate for 20 min at 37 °C before pharmacological stimulation. Subsequently, cells were exposed to the indicated treatments, and fluorescence was assessed at the time points specified in the corresponding figure legends.

Fluorescence images were acquired using a fluorescence microscope under identical acquisition settings across all experimental groups. Fluorescence intensity was quantified using Image-Pro Plus 6.0 software (Minneapolis, MN, United States). For each independent biological experiment, 5 predefined fields were analyzed under identical imaging conditions, and the mean fluorescence intensity of the analyzed fields was used to generate one value for each biological replicate. Image acquisition and quantitative analysis were performed by investigators blinded to group allocation.

### 2.8 Wound healing assay

Cells were cultured to confluence and serum-starved for 12 h before scratching. Parallel wounds were generated using a sterile pipette tip, and detached cells were removed by washing with PBS. Cells were subsequently maintained in serum-free medium containing the indicated treatments. Images were acquired at 0 and 18 h, and wound closure was quantified using ImageJ v2.1.4.7 software (National Institutes of Health, Bethesda, MD, United States).

### 2.9 Cell invasion assay

Matrigel-coated Transwell chambers were used to assess cell invasion. Cells suspended in medium containing 10% FBS were seeded into the upper chamber, and medium containing 20% FBS was added to the lower chamber as a chemoattractant. After 24 h, non-invading cells were removed, and cells that had traversed the Matrigel-coated membrane were fixed, stained with crystal violet, and quantified.

### 2.10 F-actin fluorescence staining

Cells were fixed with 4% paraformaldehyde, permeabilized with 0.1% Triton X-100, and incubated with fluorescently labeled Phalloidin (IF488-Phalloidin) for 30–120 min at room temperature in the dark. Nuclei were optionally counterstained with DAPI. Images were acquired using a fluorescence microscope following anti-fade mounting.

### 2.11 RNA extraction and quantitative real-time PCR

Total RNA was extracted from cultured cells using an RNA extraction reagent (Servicebio, Wuhan, China) according to the manufacturer’s instructions. RNA concentration and purity were measured, and equal amounts of total RNA were reverse-transcribed into complementary DNA (cDNA) using a reverse-transcription kit (Servicebio, Wuhan, China).

Quantitative real-time PCR (RT-qPCR) was performed using SYBR Green Master Mix (Servicebio, Wuhan, China) on an Applied Biosystems real-time PCR system (Thermo Fisher Scientific, Waltham, MA, United States). Relative gene expression was normalized to *TUBB* and calculated using the 2^−ΔΔCT^ method. The primer sequences were as follows: *LGALS1*, forward 5′-TCT​CAA​ACC​TGG​AGA​GTG​CC-3′ and reverse 5′-GTT​GTT​GCT​GTC​TTT​GCC​CA-3′; and *TUBB*, forward 5′-GGG​ATG​CCA​TGC​CCT​AGA​AC-3′ and reverse 5′-CTT​CAG​AGT​GCG​GAA​GCA​GA-3′.

### 2.12 Measurement of secreted Galectin-1

Galectin-1 concentrations in cell-culture supernatants were measured using a Human Galectin-1 ELISA Kit (RayBiotech, Cat. No. ELH-Galectin1; Peachtree Corners, GA, United States) according to the manufacturer’s instructions. Conditioned media were collected after the indicated treatments and centrifuged at 1,000 × *g* for 10 min at 4 °C to remove cellular debris. The resulting supernatants were used for ELISA analysis. Absorbance was measured at 450 nm using a microplate reader, and Galectin-1 concentrations were calculated from the standard curve generated with the standards supplied with the kit. Results were expressed as ng/mL.

### 2.13 Western blotting

Total protein was extracted from tissues or cells, quantified using a bicinchoninic acid assay, denatured, and separated by sodium dodecyl sulfate–polyacrylamide gel electrophoresis (SDS-PAGE). Proteins were transferred to polyvinylidene difluoride (PVDF) membranes, blocked with 5% non-fat milk, and incubated overnight at 4 °C with the following primary antibodies: Piezo1 (Abcam, Cat. No. ab327129, 1:1,000), phospho-FAK (Tyr397) (Cell Signaling Technology, Cat. No. 3283, 1:1,000), FAK (Cell Signaling Technology, Cat. No. 3285, 1:1,000), E-cadherin (Abcam, Cat. No. ab40772, 1:1,000), Vimentin (Abcam, Cat. No. ab92547, 1:2,000), YAP1 (Abcam, Cat. No. ab205270, 1:1,000), Lamin B1 (Abcam, Cat. No. ab16048, 1:1,000), Galectin-1 (Abcam, Cat. No. ab138513, 1:5,000), and β-tubulin (Abcam, Cat. No. ab6046, 1:1,000). After incubation with HRP-conjugated secondary antibodies, signals were detected by enhanced chemiluminescence. Band intensities were quantified using ImageJ v2.1.4.7 software (National Institutes of Health, Bethesda, MD, United States).

### 2.14 Generation of GCaMP6f-Expressing SCC-9 cells and xenograft fluorescence imaging

SCC-9 cells stably expressing GCaMP6f were generated by lentiviral transduction using a GCaMP6f-expressing vector (pMOS008, Addgene plasmid #163045, Watertown, MA, United States). Cells were transduced at a multiplicity of infection (MOI) of 10 in the presence of 8 μg/mL polybrene. After 24 h, the viral medium was replaced with fresh complete medium, and cells were selected with 5 μg/mL blasticidin for 7 days. Stable GCaMP6f expression was confirmed by fluorescence microscopy before xenograft establishment.

At the experimental endpoint, xenograft tumors were embedded in O.C.T. compound and prepared as 8-μm frozen sections. Native GCaMP6f fluorescence was imaged using a fluorescence microscope with identical acquisition settings across groups. Five predefined viable, non-necrotic fields per tumor were quantified after background subtraction using ImageJ software, and the mean value was used as one biological replicate per animal. Quantitative analyses were performed by investigators blinded to treatment allocation. GCaMP6f fluorescence was used as an endpoint measure of calcium-associated fluorescence; no dynamic or time-lapse calcium imaging was performed.

### 2.15 Animal experiments

Male BALB/c nude mice aged 4–6 weeks were purchased from GemPharmatech (Nanjing, China) and maintained under controlled temperature and humidity with free access to food and water. All animal procedures were approved by the Institutional Ethics Committee of Jiangxi Cancer Hospital (approval no. 2025ky301) and conducted in accordance with the Guide for the Care and Use of Laboratory Animals.

SCC-9 cells (1 × 10^6^ cells per mouse) were subcutaneously injected into donor mice. Tumor length and width were measured using digital calipers, and tumor volume was calculated as follows: volume = length × width^2^/2, where length represents the longest tumor diameter and width represents the perpendicular diameter. Tumor volume was expressed in mm^3^. When tumors reached approximately 500 mm^3^, they were excised, divided into approximately 3 × 3 × 3 mm fragments, and implanted into recipient mice. Mice bearing tumors of approximately 100 mm^3^ were enrolled in two independent experiments using separate animal cohorts.

In Experiment 1, designed to evaluate the *in vivo* effects of pharmacological modulation of Piezo1-associated mechanosensitive channel activity, mice were randomized into vehicle, Yoda1 (5 mg/kg/day, i. p.) (Wasi et al., 2025), and GsMTx4 (1 mg/kg/day, i. p.) (Yang K. et al., 2023) groups (n = 6 per group). Data from this experiment are presented in Figure 4.

In Experiment 2, conducted as a therapeutic proof-of-concept study, mice were independently randomized into vehicle, GsMTx4 (1 mg/kg/day, i. p.), OTX008 (10 mg/kg/day, i. p.) (Koonce et al., 2017), and GsMTx4 plus OTX008 groups (n = 6 per group). Data from this experiment are presented in Figure 8. No animals or data were shared between the two experiments.

Treatments were administered intraperitoneally once daily for 28 days. Tumor volume and body weight were recorded every 3 days, and animals were monitored for treatment-related toxicity. A maximum tumor diameter of 20 mm in any dimension was predefined as the maximum allowable tumor burden and one of the humane endpoints. Animals were euthanized when any tumor dimension reached 20 mm, or earlier if tumor ulceration, necrosis, impaired mobility, marked deterioration in general condition, or other signs of significant distress were observed. Ear-tagged mice were randomized using a random-number table after tumors reached the prespecified enrollment volume. Tumor measurements and quantitative tissue analyses were performed by investigators blinded to treatment allocation. Yoda1, GsMTx4, and OTX008 stock solutions prepared in DMSO were diluted with 20% sulfobutylether-β-cyclodextrin in 0.9% saline. The final DMSO and vehicle concentrations and injection volumes were identical across groups, and vehicle-treated mice received the same formulation without active compounds. No animals were excluded after randomization. At the scheduled endpoint or upon reaching the humane endpoint, mice were euthanized and tumors were collected for subsequent analyses.

### 2.16 Immunohistochemistry and immunofluorescence

Tumor tissues were fixed in 4% paraformaldehyde, embedded in paraffin, and sectioned at 4–6 μm. Sections were subjected to antigen retrieval and incubated with primary antibodies against Ki67 (Abcam, Cat. No. ab16667, 1:200), E-cadherin (Abcam, Cat. No. ab40772, 1:500), Vimentin (Abcam, Cat. No. ab92547, 1:500), or Galectin-1 (Abcam, Cat. No. ab138513, 1:1,000), followed by HRP-conjugated secondary antibodies and 3,3′-diaminobenzidine (DAB) detection. For CD31 immunofluorescence, sections underwent antigen retrieval followed by incubation with an anti-CD31 primary antibody (Abcam, Cat. No. ab182981, 1:1,000) and a fluorescent secondary antibody, and were imaged by fluorescence microscopy using identical acquisition settings across all treatment groups. For each tumor, five predefined histologically viable, non-necrotic tumor fields were analyzed. After background subtraction, CD31 fluorescence intensity was quantified using Image-Pro Plus (IPP) software (Media Cybernetics, Rockville, MD, United States). Measurements from the five fields were averaged to generate a single value for each animal and are expressed in arbitrary units (A.U.). Field selection and quantitative analysis were performed by investigators blinded to treatment allocation.

### 2.17 Statistical analysis

All quantitative data are presented as mean ± standard deviation (SD). Statistical analyses were performed using SPSS 20.0 (IBM, Armonk, NY, United States). Comparisons between two independent groups were performed using two-tailed unpaired Student’s *t*-tests, whereas paired *t*-tests were used for matched tumor and adjacent tissue samples. For comparisons among three or more groups, one-way analysis of variance (ANOVA) was performed. Homogeneity of variance was assessed before ANOVA; Tukey’s *post hoc* test was used when variances were homogeneous, whereas Tamhane’s T2 test was used when variances were unequal. Longitudinal tumor-growth data were analyzed using repeated-measures ANOVA or linear mixed-effects models, as specified in the corresponding figure legends, with treatment, time, and their interaction considered in the analysis. For xenograft experiments, each animal was treated as an independent biological replicate. Kaplan–Meier survival curves were compared using the log-rank test. Correlations between continuous variables were assessed using Pearson’s correlation coefficient. All tests were two-sided, and *P* < 0.05 was considered statistically significant.

## 3 Results

### 3.1 Piezo1 is upregulated in HNSCC

**FIGURE 1:**
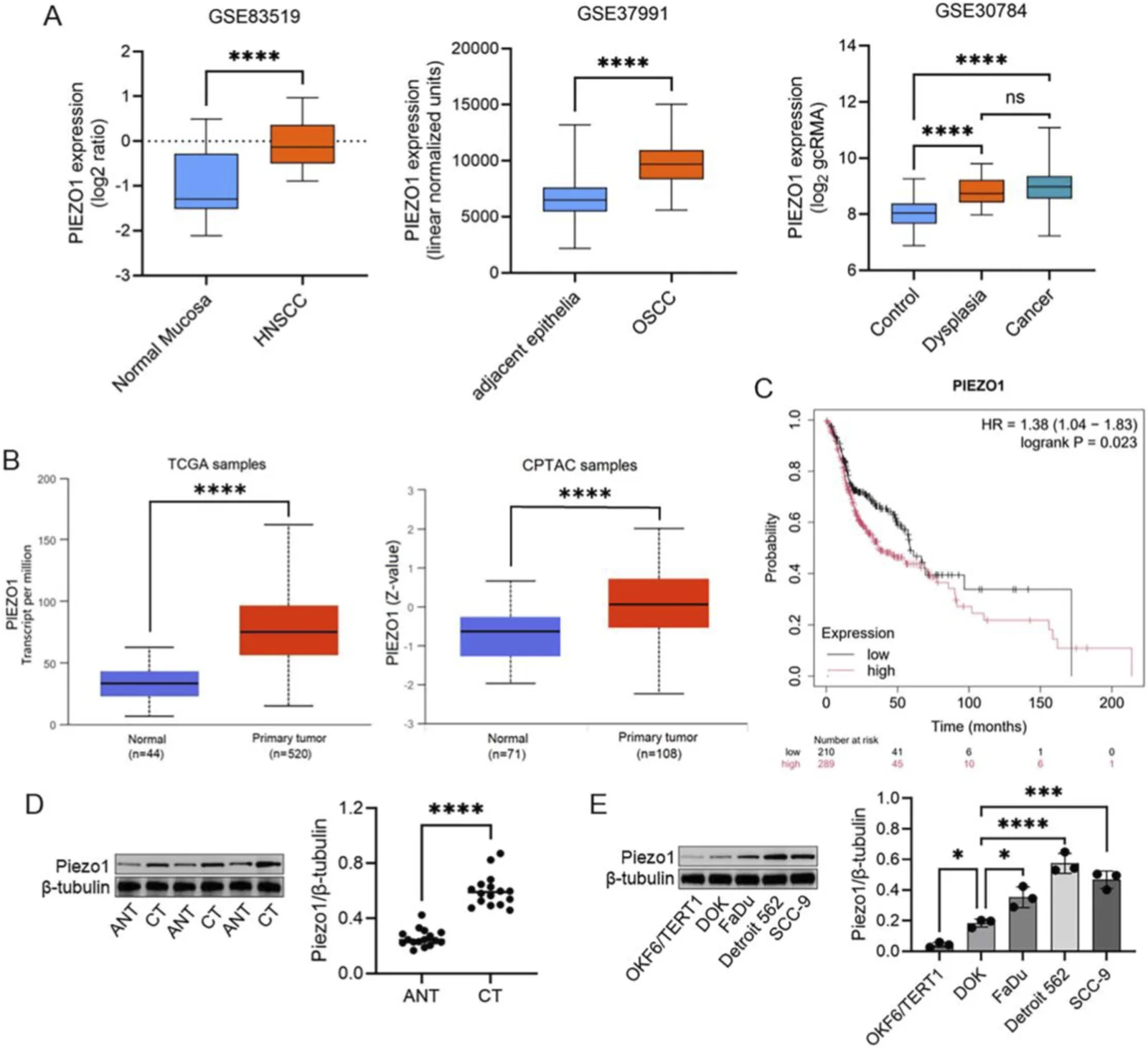
Elevated *PIEZO1* mRNA and Piezo1 protein expression in HNSCC. **(A)** *PIEZO1* mRNA expression in HNSCC based on GSE83519, GSE37991, and GSE30784. GSE83519 comprised 22 paired HNSCC and adjacent normal mucosal samples, whereas GSE37991 comprised 40 paired OSCC and adjacent normal tissue samples. GSE30784 included normal oral mucosa, dysplastic lesions, and OSCC tissues. **(B)** Comparison of *PIEZO1* expression between normal and primary tumor tissues in the TCGA cohort (normal, n = 44; primary tumor, n = 520) and between normal and primary tumor tissues in the CPTAC cohort (normal, n = 71; primary tumor, n = 108). **(C)** Kaplan-Meier analysis of overall survival in patients with HNSCC stratified into high- and low-*PIEZO1* expression groups using the median expression value as the cutoff. The hazard ratio and log-rank *P* value are shown in the panel. **(D)** Representative Western blots and densitometric quantification of Piezo1 protein in 16 paired OSCC tumor tissues (CT) and adjacent non-tumor tissues (ANT). Each point represents one patient pair. **(E)** Representative Western blots and densitometric quantification of Piezo1 protein in OKF6/TERT1, DOK, FaDu, Detroit 562, and SCC-9 cells. Data in **(D,E)** are presented as mean ± SD; n = 16 patient pairs in **(D)** and n = 3 independent biological experiments in **(E)**. Paired datasets and paired clinical tissues were analyzed using a two-tailed paired Student’s t-test; comparisons among three or more groups were performed using one-way ANOVA followed by Tukey’s or Tamhane’s T2 *post hoc* test, as appropriate. Survival differences were assessed using the log-rank test. ^*^ *P* < 0.05, ^***^ *P* < 0.001, and ^****^ *P* < 0.0001.

Analysis of public datasets revealed elevated PIEZO1 expression in HNSCC. GEO datasets GSE83519 and GSE37991 showed higher PIEZO1 mRNA levels in tumor tissues than in the corresponding normal controls. In GSE30784, which included normal mucosa, premalignant lesions, and tumor tissues, PIEZO1 expression was significantly increased in both premalignant and malignant samples compared with normal mucosa. Analyses of TCGA transcriptomic data and CPTAC proteomic data likewise showed higher PIEZO1/Piezo1 expression in HNSCC tissues than in normal tissues (Figures 1A,B).

In the primary Kaplan-Meier analysis, high PIEZO1 expression was associated with shorter overall survival (HR = 1.38, *P* = 0.023) (Figure 1C). However, the corresponding analyses using UALCAN and GEPIA showed non-significant trends toward shorter overall survival in the high-PIEZO1 groups (Supplementary Figure S2). Thus, the association between PIEZO1 expression and survival was modest and was not uniformly reproduced across the different analytical platforms.

In 16 paired OSCC specimens, Piezo1 protein abundance was significantly higher in tumor tissues than in adjacent non-tumor tissues (Figure 1D). Piezo1 protein was also elevated in premalignant DOK cells and in the HNSCC cell lines FaDu, Detroit 562, and SCC-9 compared with immortalized normal oral epithelial OKF6/TERT1 cells (Figure 1E). Collectively, these findings consistently support the upregulation of Piezo1 in HNSCC and OSCC experimental models, whereas its prognostic relevance remains exploratory and requires validation in independent, clinically annotated cohorts with multivariable adjustment.

### 3.2 Genetic and pharmacological modulation of Piezo1-Associated signaling alters EMT-Related phenotypes in HNSCC cells *in vitro*

**FIGURE 2:**
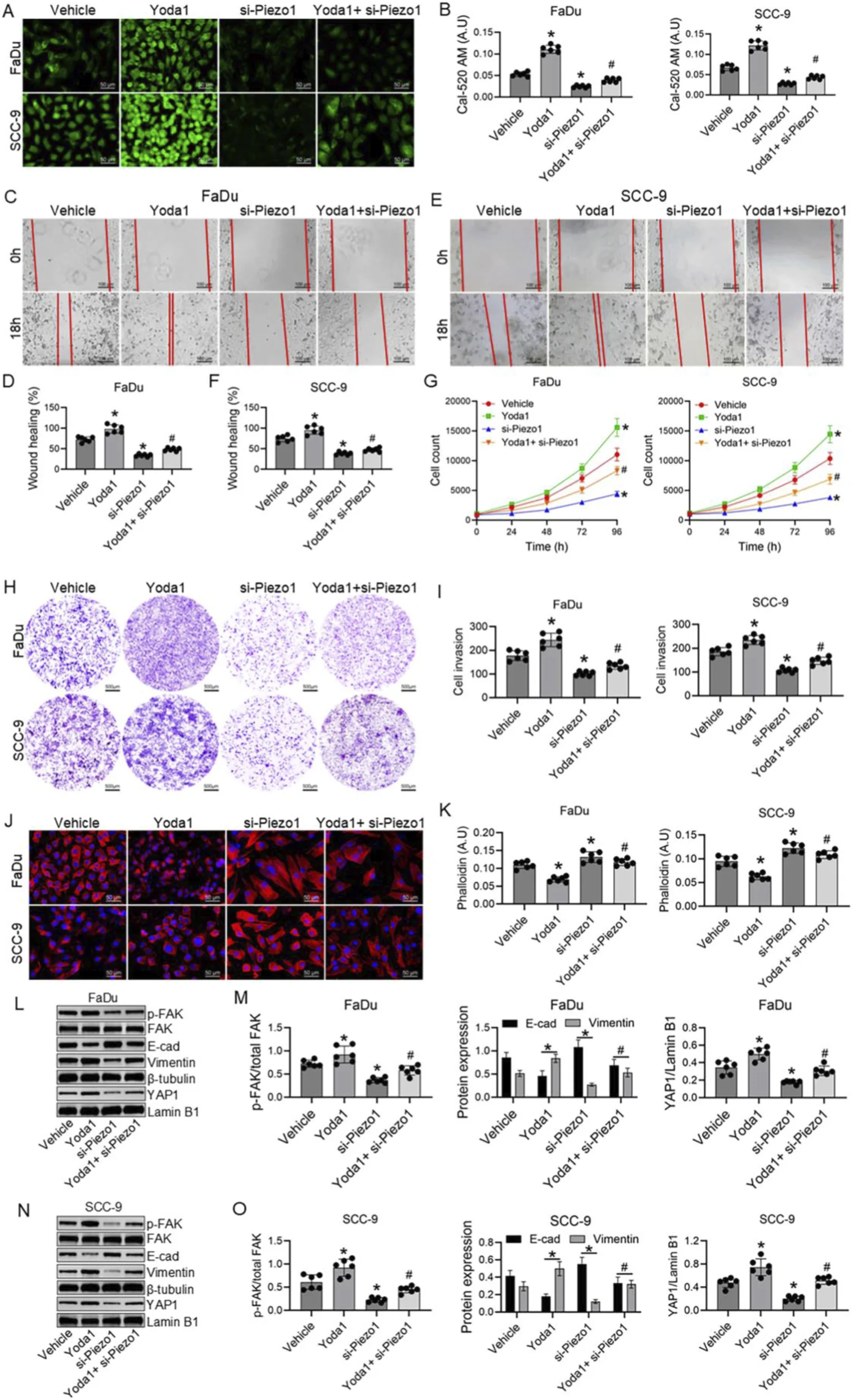
*PIEZO1* knockdown attenuates Piezo1-associated calcium signals and EMT-related phenotypes in HNSCC cells. FaDu and SCC-9 cells were assigned to the vehicle, Yoda1 (10 μM), si-Piezo1, and Yoda1 (10 μM) plus si-Piezo1 groups. For experiments involving Piezo1 silencing, the corresponding control cells were transfected with a non-targeting control siRNA (si-Ctrl). **(A,B)** Representative Cal-520 AM fluorescence images and quantification of intracellular Ca^2+^-associated fluorescence. Cells were loaded with Cal-520 AM (5 μM) for 60 min and subsequently exposed to vehicle or Yoda1 (10 μM) for 15 min before imaging. Scale bars, 50 μm. **(C–F)** Representative wound-healing images acquired at 0 and 18 h and quantification of wound closure in FaDu and SCC-9 cells. Scale bars, 100 μm. **(G)** Cell-proliferation curves recorded over 96 h **(H,I)** Representative crystal-violet-stained images and quantification of cells that traversed the Matrigel-coated Transwell membrane after 24 h. Scale bars, 500 μm. **(J,K)** Representative phalloidin staining of F-actin and quantification of fluorescence intensity after 24 h. Scale bars, 50 μm. (L–O) Representative Western blots and densitometric analyses of p-FAK/total FAK, E-cadherin, Vimentin, and nuclear YAP1 normalized to Lamin B1 in FaDu and SCC-9 cells after 24 h. Data are presented as mean ± SD from six independent biological experiments (n = 6); n refers to independently cultured samples rather than technical fields or wells. Representative images are shown, and image-based measurements were quantified from the independent experiments under identical acquisition settings. Quantification was performed by investigators blinded to group allocation. Proliferation curves were analyzed using two-way repeated-measures ANOVA with treatment, time, and treatment-by-time interaction. Other comparisons were performed using one-way ANOVA followed by Tukey’s or Tamhane’s T2 *post hoc* test, as appropriate. ^*^ *P* < 0.05 versus vehicle; ^#^ *P* < 0.05 vs. Yoda1.

To assess Piezo1-associated Ca^2+^ signaling, intracellular Ca^2+^-associated fluorescence was measured using Cal-520 AM. Yoda1 treatment significantly increased Cal-520 fluorescence in FaDu and SCC-9 cells, whereas *PIEZO1* knockdown reduced basal fluorescence and attenuated the Yoda1-induced increase (Figures 2A,B). Functionally, Yoda1 increased wound closure, proliferation, and invasion, while *PIEZO1* silencing suppressed these behaviors and attenuated Yoda1-induced effects (Figures 2C–I).

Cytoskeletal staining revealed that Yoda1 altered F-actin organization, while *PIEZO1* knockdown partially restored the actin-stress-fiber pattern (Figures 2J,K). Western blotting confirmed that Yoda1 decreased E-cadherin and increased p-FAK and Vimentin, consistent with EMT-related molecular changes. Conversely, *PIEZO1* silencing attenuated these changes (Figures 2L,M). Nuclear YAP1 levels were elevated by Yoda1 but suppressed by *PIEZO1* knockdown, supporting an association between Piezo1 modulation and YAP1-associated signaling.

**FIGURE 3:**
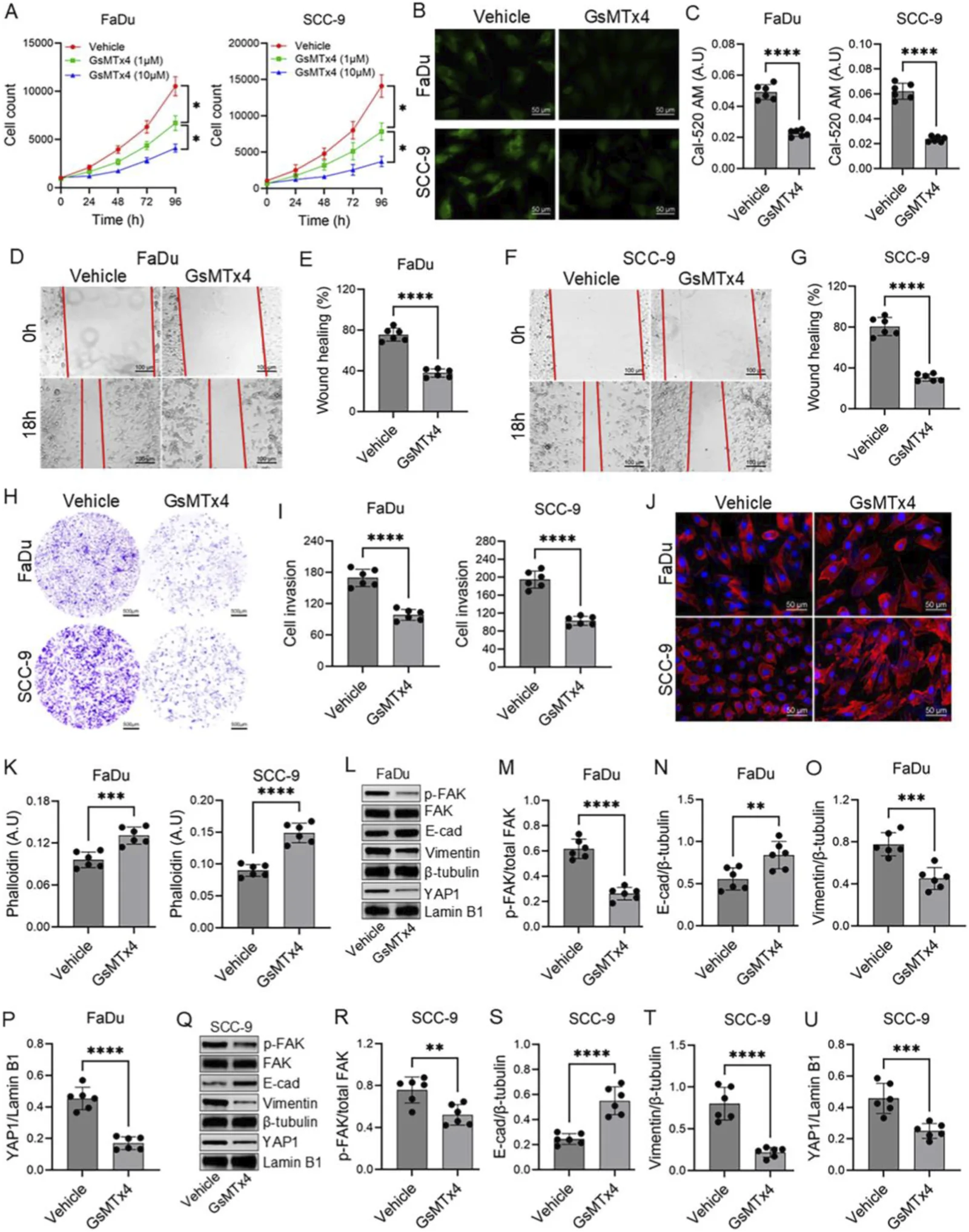
GsMTx4 attenuates EMT-related phenotypes in HNSCC cells *in vitro*. FaDu and SCC-9 cells were treated with vehicle or GsMTx4. For the proliferation assay, GsMTx4 was used at 1 and 10 μM; GsMTx4 was used at 10 μM for the remaining experiments. **(A)** Cell-proliferation curves recorded over 96 h **(B,C)** Representative Cal-520 AM fluorescence images and quantification of intracellular Ca^2+^-associated fluorescence. Cells were loaded with Cal-520 AM (5 μM) for 60 min and subsequently treated with GsMTx4 (10 μM) for 15 min before imaging. Scale bars, 50 μm. **(D–G)** Representative wound-healing images acquired at 0 and 18 h and quantification of wound closure in FaDu and SCC-9 cells. Scale bars, 100 μm. **(H,I)** Representative crystal-violet-stained images and quantification of cells that traversed the Matrigel-coated Transwell membrane after 24 h of GsMTx4 treatment. Scale bars, 500 μm. **(J,K)** Representative phalloidin staining of F-actin and quantification of fluorescence intensity after 24 h of GsMTx4 treatment. Scale bars, 50 μm. **(L–U)** Representative Western blots and densitometric analyses of p-FAK/total FAK, E-cadherin, Vimentin, and nuclear YAP1 normalized to Lamin B1 in FaDu and SCC-9 cells after 24 h of GsMTx4 treatment. Data are presented as mean ± SD from six independent biological experiments (n = 6); n denotes independently cultured biological samples. Representative images are shown, and image-based measurements were quantified from independent experiments under identical acquisition settings. Quantification was performed by investigators blinded to group allocation. Proliferation curves were analyzed using two-way repeated-measures ANOVA. Endpoint comparisons were analyzed using a two-tailed unpaired Student’s t-test. ^*^ *P* < 0.05, ^**^ *P* < 0.01, ^***^ *P* < 0.001, and ^****^ *P* < 0.0001.

Pharmacological inhibition of mechanosensitive channel activity with GsMTx4 further supported these findings. GsMTx4 dose-dependently inhibited proliferation (Figure 3A), significantly reduced intracellular Ca^2+^-associated fluorescence (Figures 3B,C), suppressed wound closure and invasion (Figures 3D–I), restored E-cadherin, reduced Vimentin, and reduced nuclear YAP1 accumulation (Figures 3J–U). The concordant molecular, functional, and phenotypic effects of *PIEZO1* silencing, together with pharmacological modulation of mechanosensitive channel activity, support an important contribution of Piezo1-associated signaling to the observed Ca^2+^-associated fluorescence changes and EMT-related phenotypes.

### 3.3 Pharmacological modulation of Piezo1-associated mechanosensitive signaling alters SCC-9 xenograft growth and EMT-Related phenotypes

**FIGURE 4:**
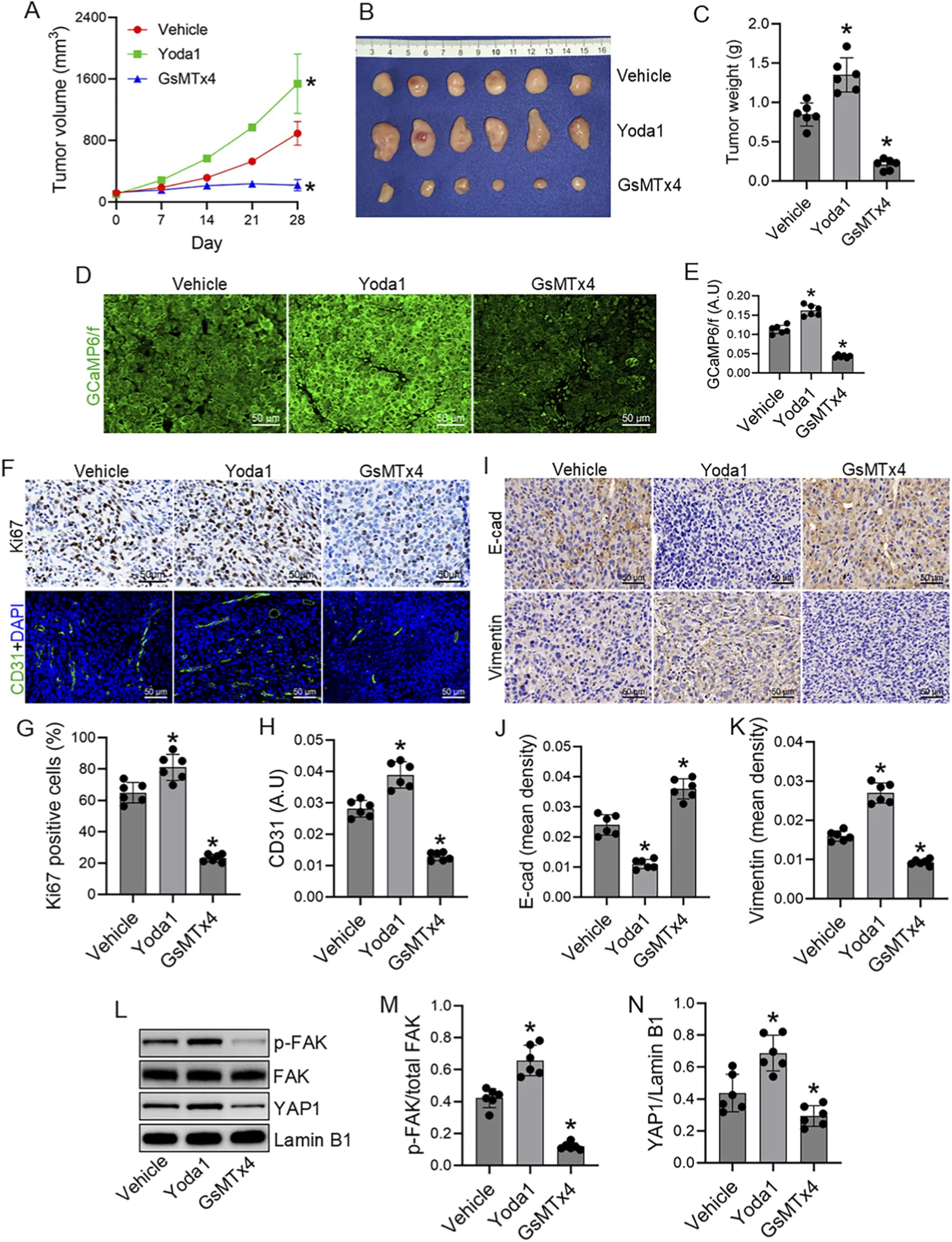
Pharmacological modulation of Piezo1-associated mechanosensitive activity alters SCC-9 xenograft growth and EMT-related phenotypes. Mice bearing SCC-9 xenografts were randomized to receive vehicle, Yoda1 (5 mg/kg/day, intraperitoneally), or GsMTx4 (1 mg/kg/day, intraperitoneally) once daily for 28 days (n = 6 mice per group). **(A)** Tumor-volume trajectories during the 28-day treatment period. **(B)** Gross morphology of excised tumors at the experimental endpoint. **(C)** Endpoint tumor weights. **(D,E)** Representative endpoint native GCaMP6f fluorescence images of frozen xenograft sections and quantification of relative fluorescence intensity. No dynamic or time-lapse calcium imaging was performed. Scale bars, 50 μm. **(F–H)** Representative Ki67 immunohistochemistry and CD31 immunofluorescence images, with quantification of Ki67-positive tumor cells and relative CD31 fluorescence intensity, respectively. Scale bars, 50 μm. **(I–K)** Representative immunohistochemical images and quantification of E-cadherin and Vimentin expression. Scale bars, 50 μm. **(L–N)** Representative Western blots and densitometric analyses of p-FAK/total FAK and nuclear YAP1 normalized to Lamin B1 in xenograft tissues. Data are presented as mean ± SD; n = 6 individual mice per group, and each point represents one animal. For tissue imaging, measurements from predefined tumor regions were averaged to generate one value per mouse. Tumor measurements and quantitative tissue analyses were performed by investigators blinded to treatment allocation. Tumor-volume trajectories were analyzed using a linear mixed-effects model with treatment, time, and treatment-by-time interaction as fixed effects and mouse as the subject-level random effect. Endpoint measurements were analyzed using one-way ANOVA followed by Tukey’s or Tamhane’s T2 *post hoc* test, as appropriate. ^*^ *P* < 0.05 vs. vehicle.

We next tested pharmacological modulation of Piezo1-associated mechanosensitive signaling *in vivo* using SCC-9 xenografts. Yoda1 treatment accelerated tumor growth, whereas GsMTx4 significantly reduced tumor progression (Figures 4A–C). Body weight remained generally stable across treatment groups during the 28-day treatment period, and no overt treatment-related toxicity was observed. Endpoint native GCaMP6f fluorescence intensity was higher in Yoda1-treated tumors and lower in GsMTx4-treated tumors than in vehicle controls (Figures 4D,E). Because GCaMP6f fluorescence was assessed in frozen tumor sections only at the experimental endpoint, these measurements represent endpoint Ca^2+^-associated fluorescence rather than dynamic Ca^2+^ influx.

Endpoint tissue analysis showed that Yoda1-treated tumors had higher Ki67 positivity and CD31 fluorescence intensity, whereas both parameters were lower in the GsMTx4-treated group (Figures 4F–H). These findings indicate differences in endpoint CD31-positive vascular staining among the treatment groups; however, because CD31 was assessed only at the experimental endpoint, they do not establish a direct effect of Yoda1 or GsMTx4 on angiogenesis. Yoda1 treatment was associated with decreased E-cadherin and increased Vimentin, whereas GsMTx4 showed the opposite pattern (Figures 4I–K). Western blotting showed increased p-FAK and nuclear YAP1 abundance following Yoda1 treatment and lower levels following GsMTx4 treatment (Figures 4L–N). Together, these findings show that pharmacological modulation of mechanosensitive channel activity was associated with differences in tumor growth, EMT-related molecular changes, endpoint Ca^2+^-associated fluorescence, and nuclear YAP1 abundance in SCC-9 xenografts, consistent with the involvement of Piezo1-associated mechanosensitive signaling.

### 3.4 Exploratory network analysis prioritizes LGALS1 for experimental validation

**FIGURE 5:**
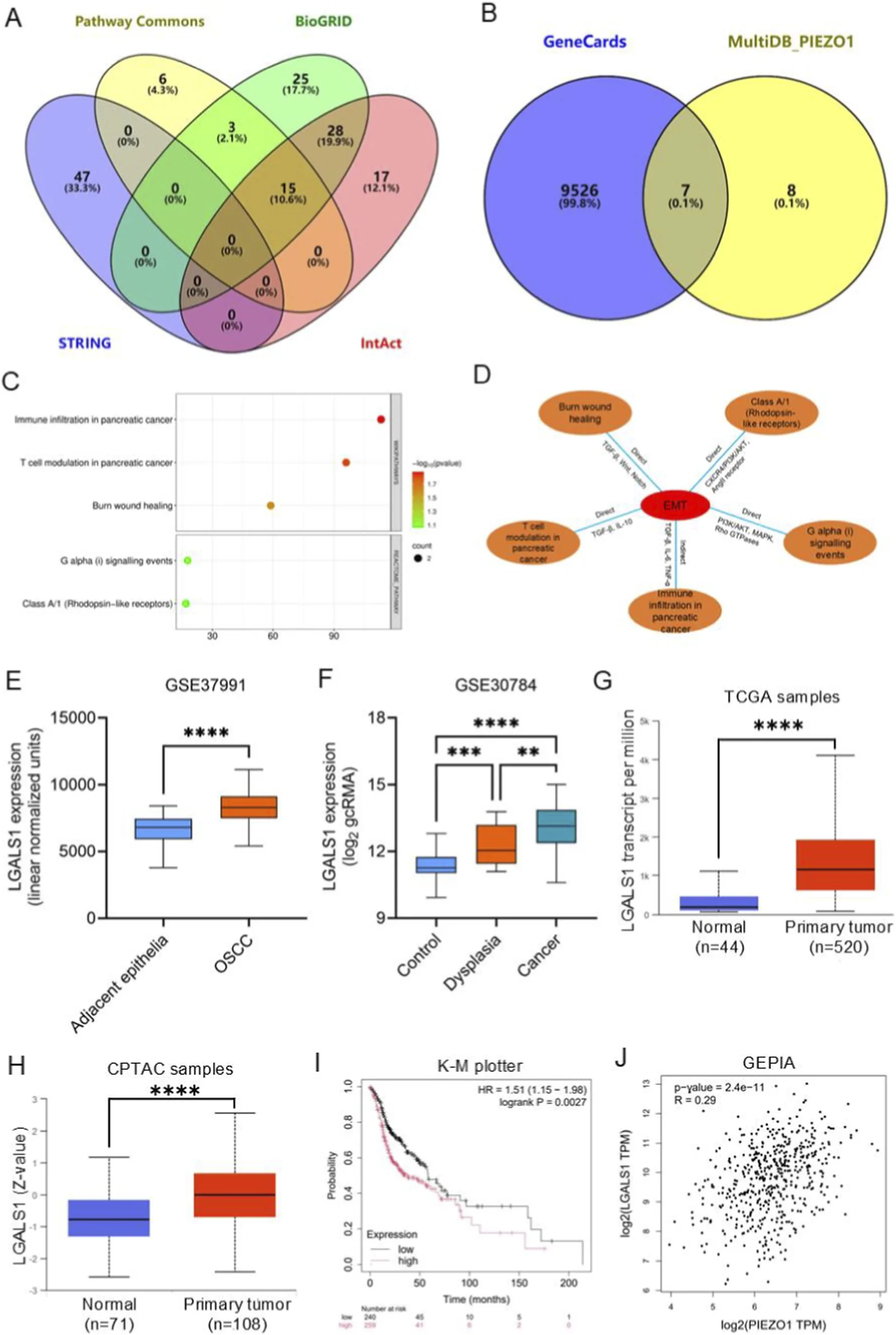
Exploratory network-based prioritization of *LGALS1* in HNSCC. **(A)** Petal diagram showing candidate genes potentially associated with *PIEZO1* retrieved from Pathway Commons, BioGRID, STRING, and IntAct. No gene was shared across all four databases. Fifteen genes were common to Pathway Commons, BioGRID, and IntAct: *VAPA, LGALS9, GPR55, SLC39A4, CYP20A1, LGALS3, FPR2, LGALS1, ATP2A1, FBXO2, ATP2A3, OR10H1, KCNK1, CLEC12B,* and *CXCR4*. **(B)** Intersection of these 15 candidate genes with a broad GeneCards-derived HNSCC gene set yielded seven overlapping genes: *CXCR4, LGALS3, LGALS1, GPR55, SLC39A4, VAPA,* and *CYP20A1*. GeneCards was used as a broad disease-relevance filter rather than as evidence of direct molecular interaction or pathway membership. **(C)** Descriptive DAVID enrichment analysis of the seven overlapping genes, visualized as a bubble plot. Because of the limited gene-set size, the enrichment analysis was used for hypothesis generation and candidate prioritization rather than pathway validation. **(D)** Schematic summary of the associations between the five enriched pathways and EMT-related processes. **(E)** *LGALS1* expression in 40 paired OSCC and adjacent normal tissues from GSE37991. **(F)** *LGALS1* expression in normal mucosa, dysplastic lesions, and OSCC tissues from GSE30784. **(G)** *LGALS1* expression in normal (n = 44) and primary HNSCC tissues (n = 520) from TCGA. **(H)** Galectin-1 protein abundance in normal (n = 71) and primary HNSCC tissues (n = 108) from CPTAC. **(I)** Exploratory Kaplan-Meier analysis of the association between *LGALS1* expression and overall survival in patients with HNSCC. **(J)** Pearson correlation between *PIEZO1* and *LGALS1* expression analyzed using GEPIA. For the expression analyses, n denotes independent patient-derived tissue samples. GSE37991 was analyzed using a paired statistical test; GSE30784 was analyzed using one-way ANOVA with the appropriate *post hoc* test; TCGA and CPTAC two-group comparisons were analyzed using two-tailed unpaired tests. Survival differences were evaluated using the log-rank test, and the association in **(J)** was evaluated using Pearson’s correlation. ^**^ *P* < 0.01, ^***^ *P* < 0.001, and ^****^ *P* < 0.0001.

To prioritize candidate genes for downstream experimental evaluation, we performed an exploratory network analysis using Pathway Commons, BioGRID, STRING, and IntAct. Because no gene was shared across all four databases, the 15 genes shared by Pathway Commons, BioGRID, and IntAct were retained and intersected with a GeneCards-derived HNSCC gene set, yielding seven candidate genes (Supplementary Figure S3, Figures 5A,B). Functional enrichment analysis of this seven-gene set using DAVID identified several EMT-related terms; however, given the limited number of genes, the enrichment results were interpreted descriptively and not as evidence of an established pathway (Figures 5C,D).

LGALS1 was prioritized for experimental validation based on its consistent upregulation across multiple HNSCC datasets, increased expression across disease progression in GSE30784, positive correlation with PIEZO1 (R = 0.29, *P* < 0.001), and established relevance to EMT (Figures 5E–J). In contrast, CXCR4 was upregulated in tumor-versus-normal comparisons but negatively correlated with PIEZO1 within the HNSCC tumor cohort (Supplementary Figures S3–S5).

**FIGURE 6:**
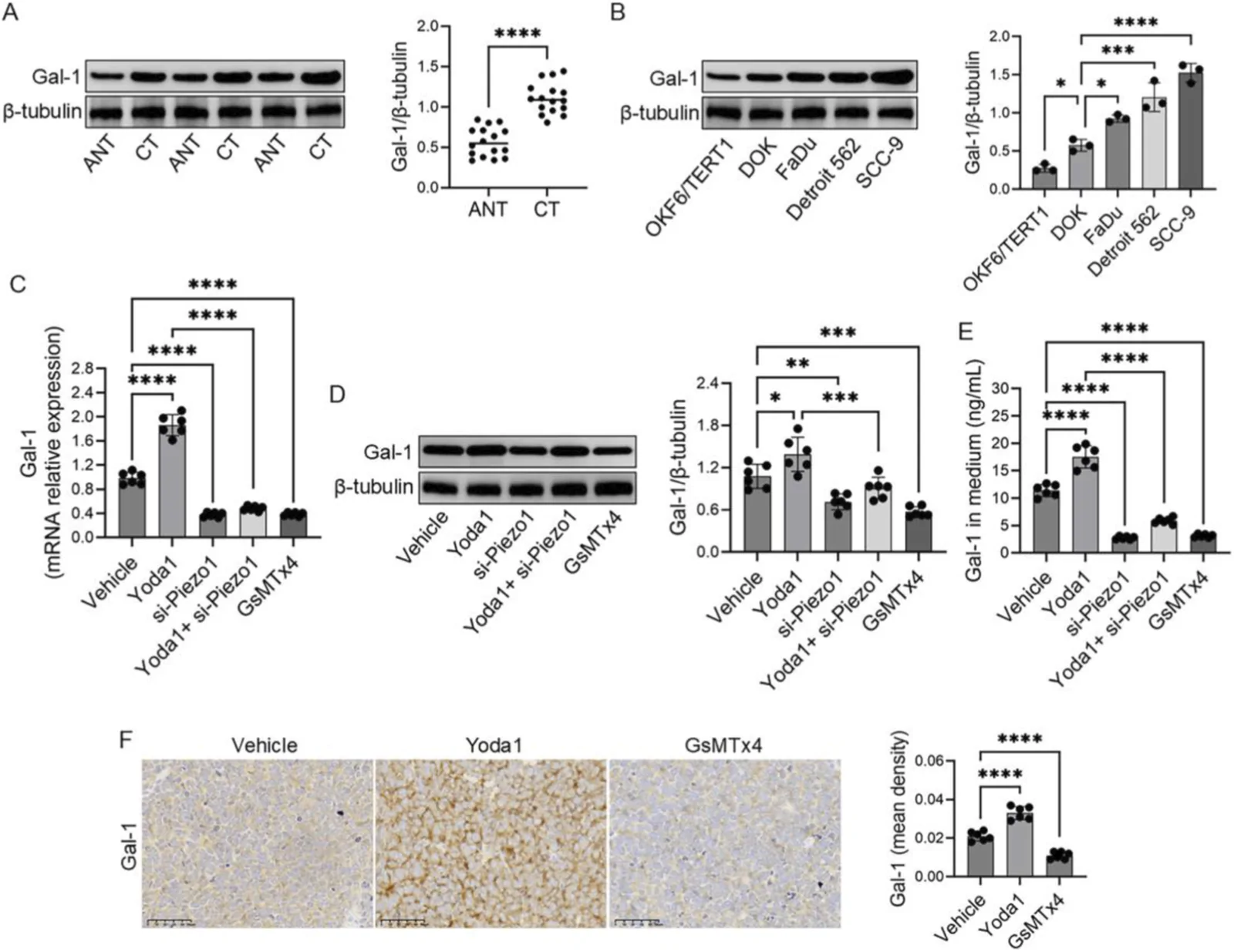
Galectin-1 expression in HNSCC and its response to modulation of PIEZO1/Piezo1-associated signaling. **(A)** Representative Western blots and densitometric quantification of Galectin-1 protein in 16 paired OSCC tumor tissues (CT) and adjacent non-tumor tissues (ANT). Each point represents one patient pair. **(B)** Representative Western blots and densitometric quantification of Galectin-1 protein in OKF6/TERT1, DOK, FaDu, Detroit 562, and SCC-9 cells. **(C–E)** Galectin-1 mRNA expression, cellular protein abundance, and secretion into the culture medium in SCC-9 cells assigned to the vehicle, Yoda1 (10 μM), si-Piezo1, Yoda1 (10 μM) plus si-Piezo1, and GsMTx4 (10 μM) groups. For comparisons involving Piezo1 silencing, the corresponding control cells were transfected with a non-targeting control siRNA (si-Ctrl). Cells were analyzed after 24 h of treatment. **(F)** Representative Galectin-1 immunohistochemistry and quantification in SCC-9 xenografts from mice treated with vehicle, Yoda1 (5 mg/kg/day), or GsMTx4 (1 mg/kg/day) for 28 days. Scale bars, 50 μm. Data are presented as mean ± SD. n = 16 patient pairs in **(A)**, n = 3 independent biological experiments in **(B)**, n = 6 independent biological experiments in **(C–E)**, and n = 6 individual mice per group in **(F)**. Quantification of xenograft sections was performed by investigators blinded to treatment allocation, with multiple fields averaged to obtain one value per mouse. Paired clinical tissues were analyzed using a two-tailed paired Student’s t-test. Comparisons among three or more groups were performed using one-way ANOVA followed by Tukey’s or Tamhane’s T2 *post hoc* test, as appropriate. Exact comparisons are indicated by brackets. ^*^ *P* < 0.05, ^**^ *P* < 0.01, ^***^ *P* < 0.001, ^****^ *P* < 0.0001.

Experimental follow-up showed that Yoda1 increased Galectin-1 expression and secretion in SCC-9 cells, whereas *PIEZO1* silencing or GsMTx4 treatment attenuated these effects. In SCC-9 xenografts, Galectin-1 expression was likewise increased following Yoda1 treatment and decreased following GsMTx4 treatment (Figure 6). These findings support Galectin-1 as a Piezo1-responsive candidate effector in the examined models but do not establish a direct physical interaction or a linear Piezo1–Galectin-1 pathway.

### 3.5 Galectin-1 is responsive to modulation of Piezo1-Associated signaling in HNSCC

**FIGURE 7:**
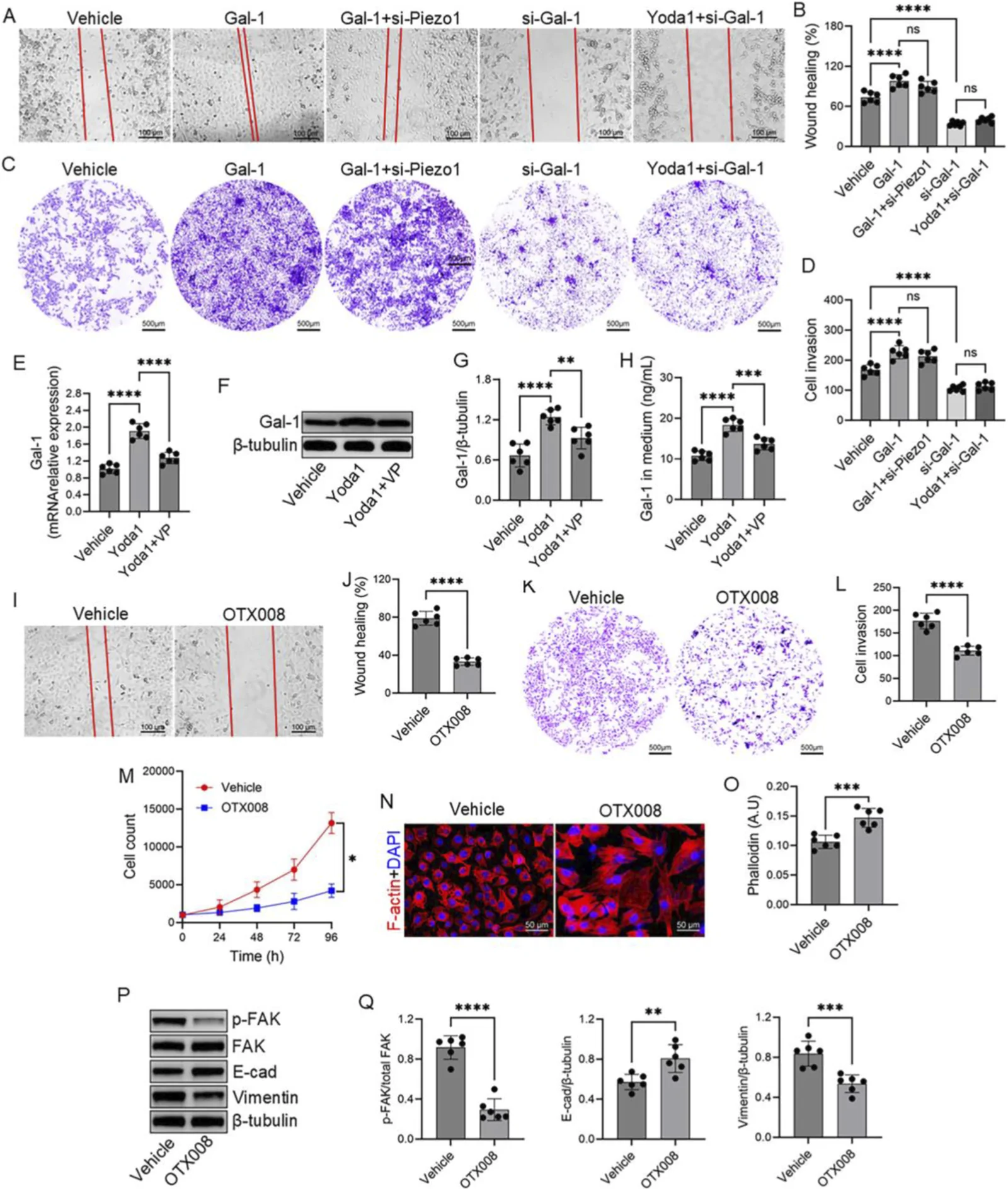
Galectin-1 modulates EMT-related phenotypes in SCC-9 cells *in vitro*. **(A–D)** SCC-9 cells were assigned to the vehicle, recombinant Galectin-1 (100 ng/mL), recombinant Galectin-1 plus si-Piezo1, si-Gal-1, and Yoda1 (10 μM) plus si-Gal-1 groups. For comparisons involving siRNA-mediated silencing, the corresponding control cells were transfected with a non-targeting control siRNA (si-Ctrl). Representative wound-healing images acquired at 0 and 18 h and quantification of wound closure are shown in **(A, B)**. Scale bars, 100 μm. Representative crystal-violet-stained images and quantification of cells that traversed the Matrigel-coated Transwell membrane after 24 h are shown in **(C, D)**. Scale bars, 500 μm. **(E–H)** Galectin-1 mRNA expression, cellular protein abundance, and secretion into the culture medium in cells treated with vehicle, Yoda1 (10 μM), or Yoda1 plus verteporfin (VP; 1 μM) for 24 h **(I–L)** Representative wound-healing images acquired at 0 and 18 h and Transwell invasion images in cells treated with vehicle or OTX008 (100 μM), with the corresponding quantitative analyses. Scale bars, 100 μm in **(I)** and 500 μm in **(K)**. **(M)** Cell-proliferation curves during 96 h of vehicle or OTX008 treatment. **(N, O)** Representative phalloidin staining and quantification of F-actin fluorescence intensity after 24 h of OTX008 treatment. Scale bars, 50 μm. **(P, Q)** Representative Western blots and densitometric analyses of p-FAK/total FAK, E-cadherin, and Vimentin after 24 h of OTX008 treatment. Data are presented as mean ± SD from six independent biological experiments (n = 6); n denotes independently cultured biological samples. Representative images were acquired under identical conditions and quantified from the independent experiments. Quantification was performed by investigators blinded to group allocation. The proliferation curve in (M) was analyzed using two-way repeated-measures ANOVA. Comparisons involving three or more groups were analyzed using one-way ANOVA followed by Tukey’s or Tamhane’s T2 *post hoc* test, whereas two-group comparisons were analyzed using a two-tailed unpaired Student’s t-test. Exact comparisons are indicated by brackets. ns, not significant; ^**^ *P* < 0.01, ^***^ *P* < 0.001, ^****^ *P* < 0.0001.

To define the functional role of Galectin-1, we examined the effects of exogenous Galectin-1 supplementation and LGALS1 knockdown in SCC-9 cells. Exogenous Galectin-1 significantly enhanced wound closure and invasion, including in PIEZO1-silenced cells (Figures 7A–D). Conversely, LGALS1 knockdown attenuated these phenotypes, and Yoda1 treatment did not restore them. These findings support a functional association between Piezo1-responsive Galectin-1 and EMT-related phenotypes in SCC-9 cells.

Yoda1 increased Galectin-1 mRNA expression, cellular protein abundance, and secretion, whereas verteporfin attenuated these effects (Figures 7E–H), consistent with the involvement of YAP1-associated signaling in the Piezo1-responsive regulation of Galectin-1. OTX008 treatment reduced wound closure, invasion, and proliferation (Figures 7I–M), altered F-actin organization (Figures 7N,O), and was associated with increased E-cadherin and decreased p-FAK and Vimentin expression (Figures 7P,Q). Together, these findings support a functional role for Galectin-1-associated signaling in EMT-related phenotypes in HNSCC cells. However, because direct pharmacological target engagement was not assessed, the effects of OTX008 cannot be attributed exclusively to inhibition of Galectin-1-mediated EMT.

### 3.6 Combined GsMTx4 and OTX008 treatment produces greater antitumor effects than either monotherapy

**FIGURE 8:**
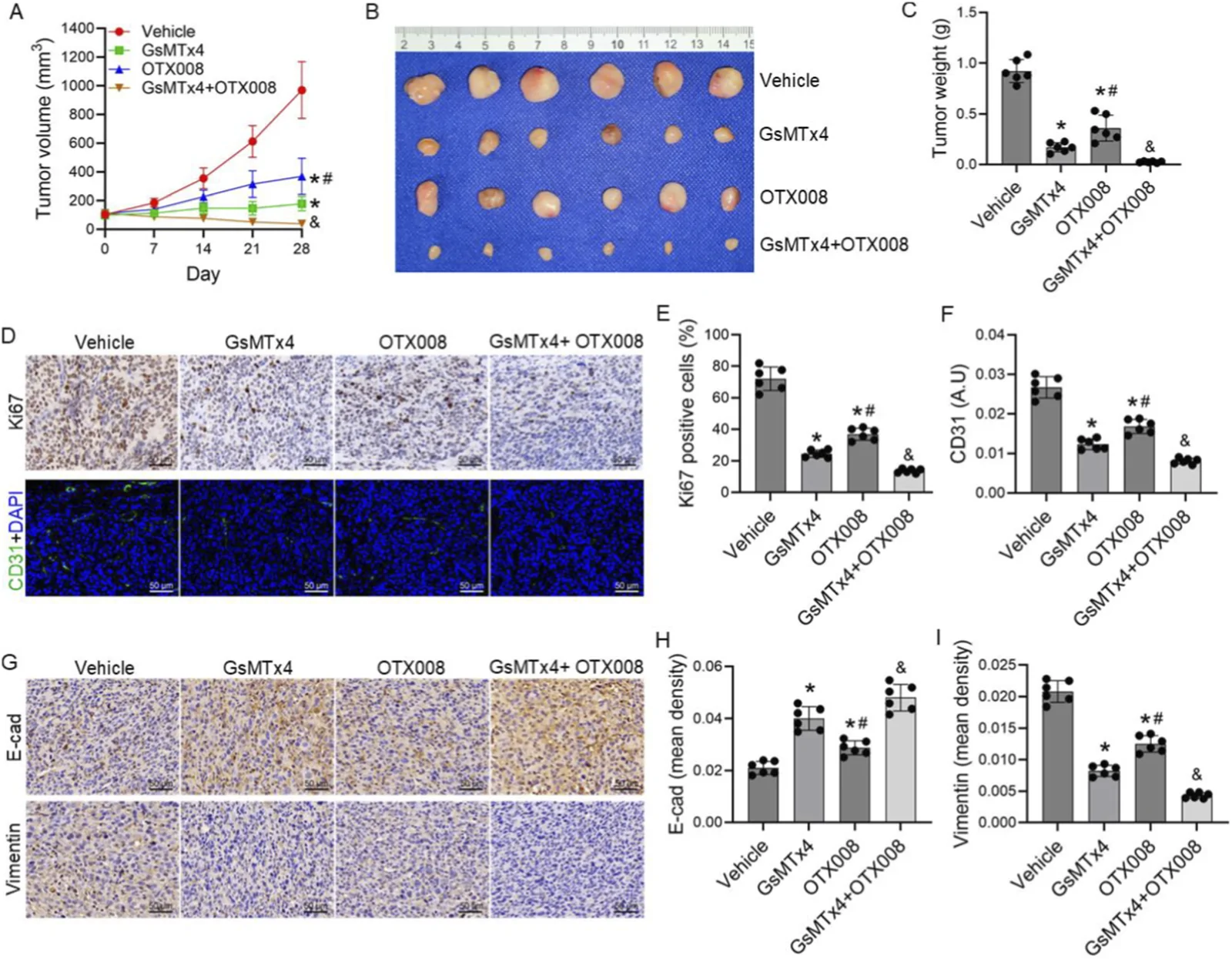
Combined GsMTx4 and OTX008 treatment produces greater antitumor effects than either monotherapy in SCC-9 xenografts. This experiment was performed using an independent cohort of SCC-9 xenograft-bearing mice. Animals were randomized to receive vehicle, GsMTx4 (1 mg/kg/day, intraperitoneally), OTX008 (10 mg/kg/day, intraperitoneally), or GsMTx4 plus OTX008 (n = 6 mice per group). All treatments were administered once daily for 28 days. **(A)** Tumor-volume trajectories during treatment. **(B)** Gross morphology of excised tumors at the experimental endpoint. **(C)** Endpoint tumor weights. **(D–F)** Representative Ki67 immunohistochemistry and CD31 immunofluorescence images, with quantification of Ki67-positive tumor cells and relative CD31 fluorescence intensity, respectively. Scale bars, 50 μm. **(G–I)** Representative immunohistochemical images and quantification of E-cadherin and Vimentin expression. Scale bars, 50 μm. Data are presented as mean ± SD; n = 6 individual mice per group, and each point represents one animal. Measurements from multiple predefined tumor fields were averaged to obtain one value per mouse. Tumor measurements and quantitative tissue analyses were performed by investigators blinded to treatment allocation. Tumor-volume trajectories were analyzed using a linear mixed-effects model with treatment, time, and treatment-by-time interaction as fixed effects and mouse as the subject-level random effect. Endpoint measurements were analyzed using one-way ANOVA followed by Tukey’s or Tamhane’s T2 *post hoc* test, as appropriate. ^*^ *P* < 0.05 vs. vehicle, ^#^ *P* < 0.05 vs. GsMTx4, ^&^ *P* < 0.05 vs. GsMTx4 or OTX008.

Finally, we evaluated combined GsMTx4 and OTX008 treatment in SCC-9 xenografts. Both GsMTx4 and OTX008 monotherapies suppressed tumor growth, but their combination produced stronger inhibition (Figures 8A–C). Body weight remained generally stable across all treatment groups during the 28-day treatment period, with no overt signs of treatment-related toxicity. Endpoint tumor analyses showed lower Ki67 positivity and CD31 fluorescence intensity following either GsMTx4 or OTX008 treatment, with lower values observed in the combination group (Figures 8D–F). Combined treatment was also associated with a greater increase in E-cadherin and decrease in Vimentin expression than either monotherapy (Figures 8G–I).

Overall, at the doses examined, combined GsMTx4 and OTX008 treatment produced greater reductions in tumor growth, Ki67 positivity, endpoint CD31 fluorescence intensity, and EMT-associated marker changes than either monotherapy. Formal pharmacological synergy was not evaluated and cannot be inferred from the present experiment.

## 4 Discussion

Ca^2+^ functions as a ubiquitous second messenger and regulates multiple processes relevant to tumor progression, including proliferation, migration, intravasation, and angiogenesis. As a mechanosensitive cation channel, Piezo1 converts mechanical cues into intracellular signals, prominently through Ca^2+^ influx. In multiple tumor contexts, aberrant Piezo1 expression or activity has been linked to malignant progression through mechanotransduction, remodeling of the tumor microenvironment, and activation of downstream oncogenic signaling (Dombroski et al., 2021; Yu and Liao, 2021). For instance, in cervical cancer, Piezo1 responds to extracellular matrix (ECM) stiffness to enhance migration and invasion (Liu et al., 2025); in gastric cancer, the Piezo1–YAP1–CTGF axis forms a positive feedback loop that drives cancer-associated fibroblast activation and collagen deposition (Chen et al., 2023); and in hepatocellular carcinoma, Piezo1-mediated Ca^2+^ influx regulates HIF1α ubiquitination and VEGF expression, promoting stiffness-driven angiogenesis (Li et al., 2022). However, Piezo1 has also been reported to exert tumor-suppressive effects in specific cancer contexts (Bo et al., 2024; O'Callaghan et al., 2022), underscoring the context-dependent nature of Piezo1 signaling in cancer.

The role of Piezo1 in HNSCC has been incompletely defined. Previous studies showed that Piezo1 is highly expressed in OSCC, is co-expressed with Ki67, and promotes calcium influx and proliferation (Hasegawa et al., 2021). Piezo1 expression also increases with ECM stiffness, correlating with increased expression of the cancer stem cell marker CD44, suggesting that it functions as a mechanosensor driving OSCC progression (Sheth et al., 2024). The YAP1/Piezo1 axis has further been implicated in cutaneous squamous cell carcinoma and HNSCC development (Yang Y. L. et al., 2023). Accordingly, the novelty of the present study does not lie in the initial association of Piezo1 with OSCC or in establishing a general connection between Piezo1 and YAP1-related signaling. Rather, the principal new finding is the identification and experimental evaluation of Galectin-1 as a functionally relevant, Piezo1-responsive candidate effector associated with EMT-related phenotypes in HNSCC. In addition, the present study provides preliminary *in vivo* proof-of-concept that concurrent pharmacological modulation of mechanosensitive channel activity and Galectin-1 produces greater antitumor effects than either intervention alone at the tested doses. Because direct YAP1/TEAD binding to the LGALS1 regulatory region was not assessed, the proposed Piezo1–YAP1–Galectin-1 relationship should be regarded as an associated signaling model rather than a fully established linear transcriptional pathway.

In this study, integrating public datasets, clinical samples, and HNSCC cell lines, we demonstrated that Piezo1 is upregulated in both precancerous and tumor tissues, whereas its association with patient survival was modest and not uniformly reproduced across analytical platforms. Functionally, Piezo1 activation via Yoda1 enhanced proliferation, wound closure, invasion, and EMT-related phenotypes in HNSCC cells, with concomitant nuclear YAP1 accumulation. Conversely, *PIEZO1* knockdown or pharmacological inhibition of mechanosensitive channel activity with GsMTx4 attenuated these effects. *In vivo*, Yoda1 accelerated xenograft tumor growth and was associated with EMT-related molecular changes and increased nuclear YAP1 abundance, whereas GsMTx4 was associated with reduced tumor growth and attenuation of these EMT-related changes. Taken together, the concordant effects of *PIEZO1* silencing and pharmacological modulation of mechanosensitive channel activity support an important contribution of Piezo1-associated signaling to Ca^2+^-associated fluorescence changes, EMT-related phenotypes, and tumor progression in the examined HNSCC models. However, because GsMTx4 is not absolutely specific for Piezo1 and the present study did not include individual-siRNA validation or genetic rescue, these findings should not be interpreted as definitive evidence of exclusive Piezo1 dependence.

Gal-1 is widely implicated in EMT across multiple cancers, though its effects depend on tumor context and microenvironmental cues. In hepatocellular carcinoma, Gal-1 downregulates E-cadherin, upregulates Snail and Vimentin, and promotes EMT via PI3K/Akt and Wnt/β-catenin signaling (Bacigalupo et al., 2015). In gastric cancer, Gal-1 enhances vasculogenic mimicry and EMT (You et al., 2019), and in triple-negative breast cancer, it drives EMT through MMP-2/9 activation (Kim et al., 2025). In HNSCC, Gal-1 overexpression correlates with poor prognosis, and its inhibition shows robust antitumor effects *in vitro* and *in vivo* (Greer et al., 2022; Koonce et al., 2017; Nambiar et al., 2023). However, the regulatory relationship between Piezo1 and Gal-1 had not been systematically explored.

In the present study, the network analysis was used as a hypothesis-generating strategy rather than as evidence of direct molecular interaction or causal pathway membership. LGALS1 was prioritized for experimental evaluation based on its reproducible upregulation across HNSCC datasets, association with disease progression, positive correlation with PIEZO1, and established relevance to EMT. Subsequent experiments showed that Galectin-1 expression and secretion were responsive to genetic silencing of PIEZO1 and pharmacological modulation of mechanosensitive channel activity. Together with the effects of Galectin-1 supplementation, knockdown, and pharmacological inhibition, these findings support Galectin-1 as a functionally relevant, Piezo1-responsive candidate effector in the examined models. However, they do not establish direct molecular regulation of LGALS1 by Piezo1 or prove that the two molecules constitute a linear signaling pathway.

Our results further showed that Galectin-1 was upregulated in HNSCC and that its expression and secretion were responsive to modulation of PIEZO1/Piezo1-associated signaling. Verteporfin attenuated the Yoda1-associated increase in Galectin-1, supporting the involvement of YAP1-associated signaling, although direct YAP1/TEAD-mediated transcriptional regulation of LGALS1 was not demonstrated. Exogenous Galectin-1 enhanced EMT-related phenotypes, whereas LGALS1 knockdown attenuated these effects. OTX008 treatment was likewise associated with reduced proliferation, wound closure, invasion, and EMT-related marker changes. Collectively, these findings support Galectin-1 as a functionally relevant, Piezo1-responsive candidate effector in the examined models. However, because direct pharmacological target engagement and rescue experiments were not performed for OTX008, its observed effects cannot be attributed exclusively to Galectin-1 inhibition or to suppression of EMT.

In the second xenograft experiment, combined administration of GsMTx4 and OTX008 produced greater tumor growth inhibition than either monotherapy at the tested doses. The combination group also showed lower Ki67 positivity and endpoint CD31 fluorescence intensity, together with increased E-cadherin and decreased Vimentin expression. These findings provide preliminary *in vivo* proof-of-concept for combined pharmacological modulation of mechanosensitive channel activity and Galectin-1-associated signaling. Importantly, CD31 fluorescence was assessed only at the experimental endpoint, when substantial differences in tumor size were already present among the treatment groups. Therefore, the observed differences in CD31-positive vascular staining may partly reflect differences in tumor burden or tissue composition and should not be interpreted as evidence that GsMTx4, OTX008, or their combination directly inhibits angiogenesis.

Several limitations should be noted. First, although pooled PIEZO1 siRNAs and GsMTx4 produced concordant effects, the individual siRNAs were not tested, rescue with siRNA-resistant PIEZO1 was not performed, and GsMTx4 is not Piezo1-specific; therefore, exclusive Piezo1 dependence cannot be established. Second, although we assessed nuclear YAP1 abundance and the effects of verteporfin, we did not directly measure YAP/TEAD transcriptional activity, evaluate TAZ, or demonstrate direct YAP1/TEAD-mediated regulation of the *LGALS1* regulatory region. Third, because proliferation was not independently blocked during the Transwell assay, treatment-related differences in cell number may have contributed to the observed invasion phenotype. Fourth, validation was limited to 16 paired OSCC specimens and a single SCC-9 xenograft model. Given the anatomical and molecular heterogeneity of HNSCC, including HPV-defined disease, and the exploratory nature of the survival analyses, the generalizability of our findings remains limited, and the independent prognostic value of PIEZO1 or LGALS1 cannot be established. Validation in larger, clinically annotated cohorts with multivariable analysis and in independent *in vivo* models is therefore required. Finally, formal pharmacological synergy between GsMTx4 and OTX008 was not evaluated.

## 5 Conclusion

Our study supports an important contribution of Piezo1-associated mechanosensitive signaling to EMT-related phenotypes and tumor progression in HNSCC models and identifies Galectin-1 as a functionally relevant, Piezo1-responsive candidate effector associated with YAP1 signaling. At the tested doses, combined GsMTx4 and OTX008 treatment produced greater antitumor effects than either monotherapy, supporting further evaluation of this co-targeting strategy. Direct YAP1-mediated transcriptional regulation of LGALS1 and formal pharmacological synergy remain to be established.

## Data Availability

The raw data supporting the conclusions of this article will be made available by the authors, without undue reservation.
