# Supplementary material for: Piezo1 promotes EMT and tumor progression in HNSCC through a YAP1-Associated Galectin-1 axis
Source: Front Cell Dev Biol. 2026 Sep 10;14:1907708. doi: 10.3389/fcell.2026.1907708 (PMC13601305; doi:10.3389/fcell.2026.1907708)
Supplement: Supplementary file 1 [file DataSheet1.pdf]

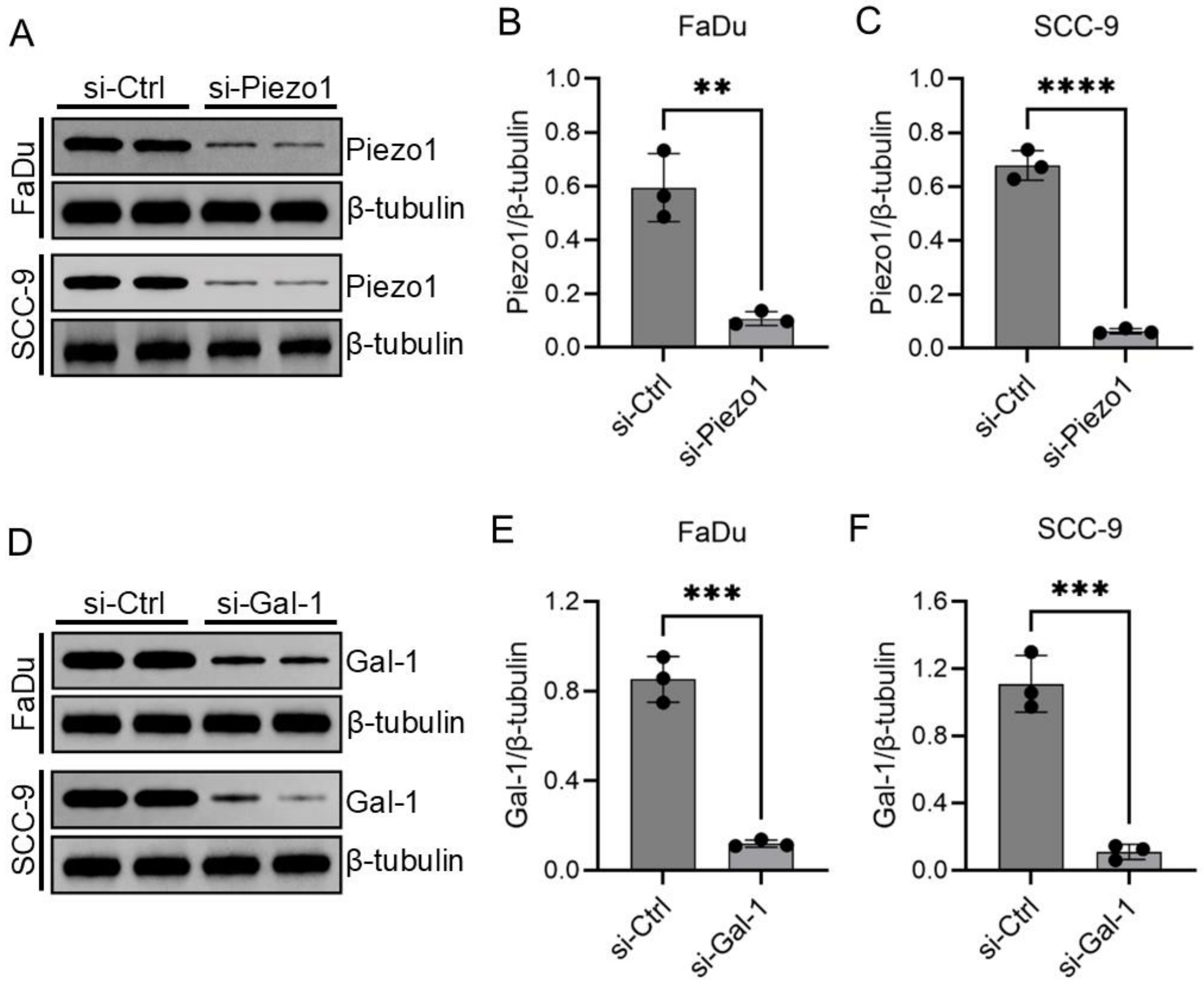

Supplementary Figure 1. Validation of *PIEZO1* and *LGALS1* knockdown in HNSCC cells. FaDu and SCC-9 cells were transfected with non-targeting control siRNA (si-Ctrl), siRNA targeting *PIEZO1* (si-Piezo1), or siRNA targeting *LGALS1* (si-Gal-1), as indicated. (A) Representative Western blots showing Piezo1 protein expression in FaDu and SCC-9 cells following *PIEZO1* silencing. (B, C) Densitometric quantification of Piezo1 protein expression normalized to β-tubulin in FaDu and SCC-9 cells, respectively. (D) Representative Western blots showing Galectin-1 (Gal-1) protein expression following *LGALS1* silencing. (E, F) Densitometric quantification of Gal-1 protein expression normalized to β-tubulin in FaDu and SCC-9 cells, respectively. Data are presented as mean ± SD from three independent biological experiments (n = 3). Statistical comparisons were performed using two-tailed unpaired Student's *t*-tests. $^{**}P < 0.01$, $^{***}P < 0.001$, and $^{****}P < 0.0001$ vs. si-Ctrl.

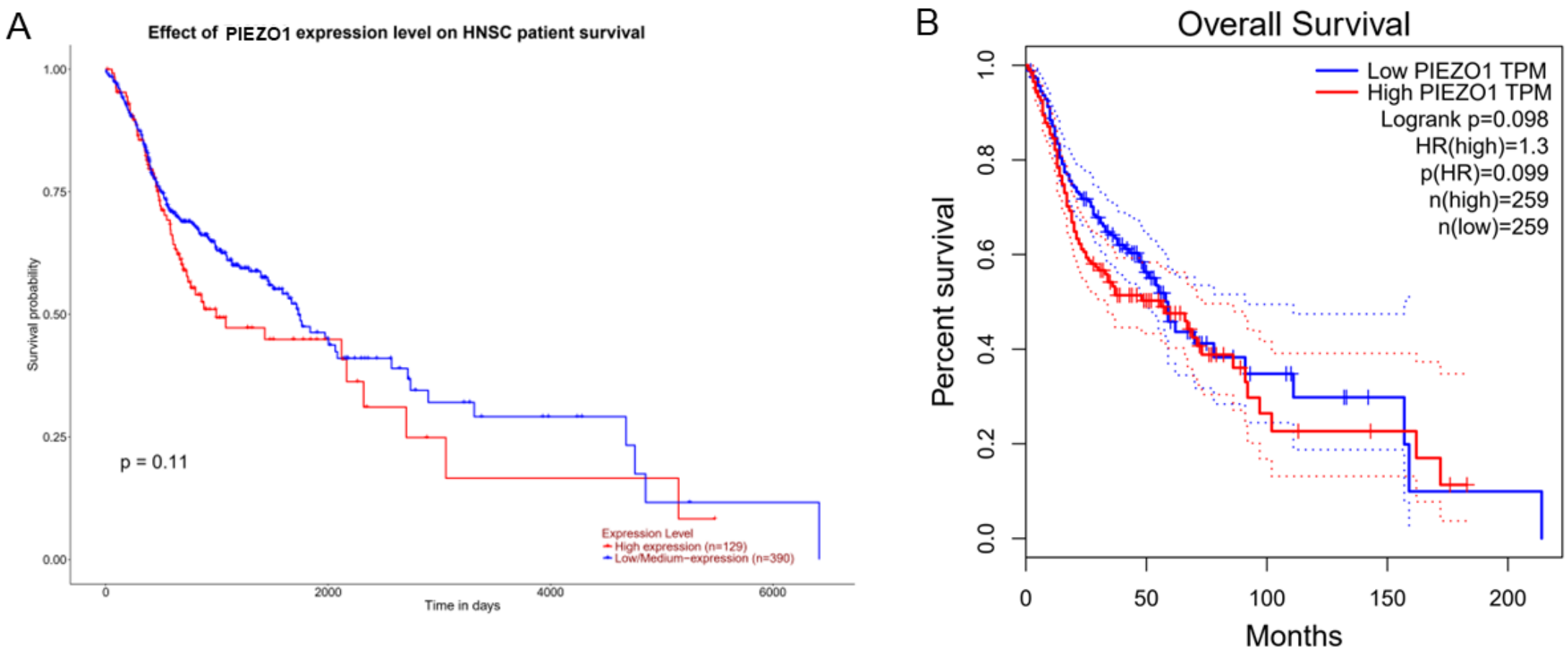

Supplementary Figure 2. Association between *PIEZO1* expression and survival in HNSCC patients. (A) Survival curves from the UALCAN database. (B) Survival curves from the GEPIA database.

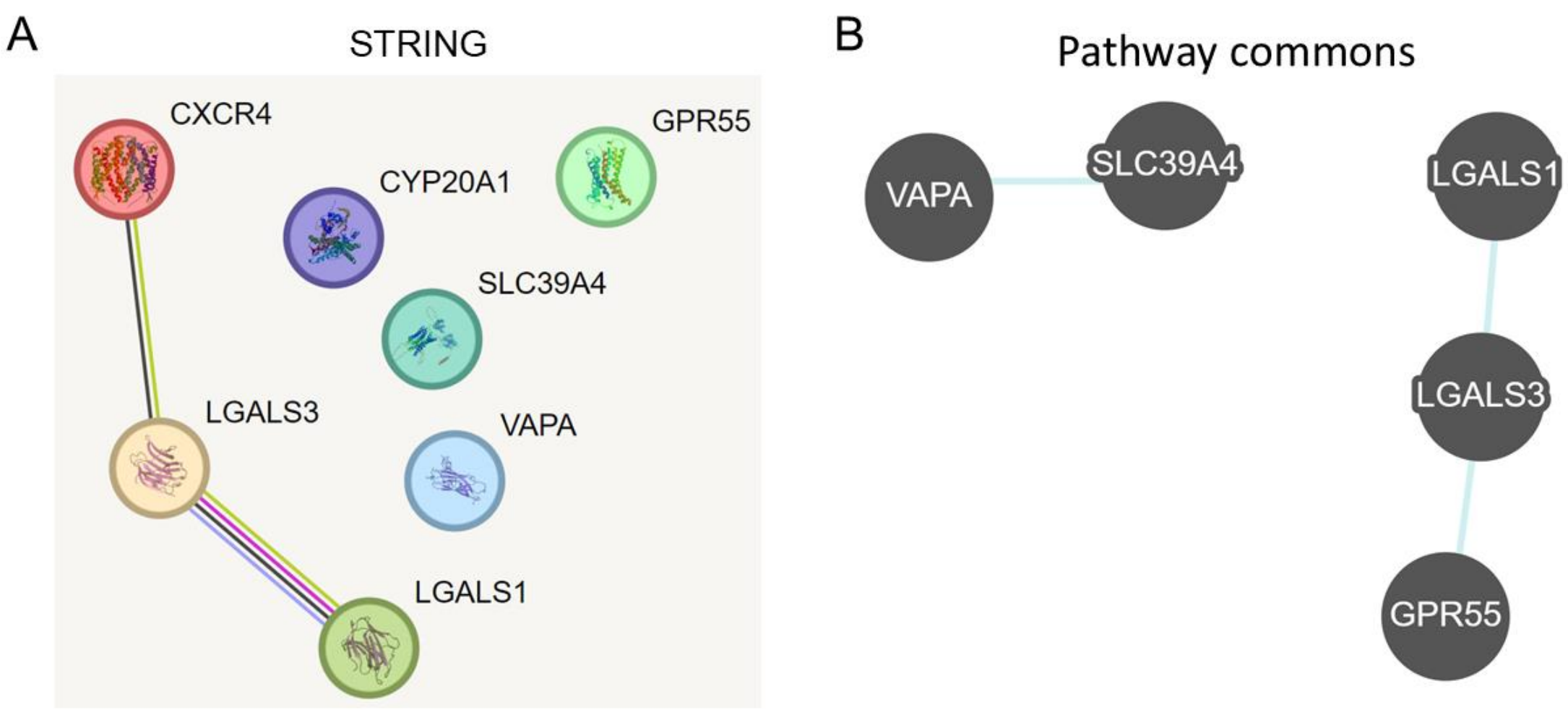

Supplementary Figure 3. Interaction analysis of overlapping genes. (A) STRING network of 7 overlapping genes (*CXCR4*, *LGALS3*, *LGALS1*, *GPR55*, *SLC39A4*, *VAPA*, *CYP20A1*), showing connections among *CXCR4*, *LGALS3*, and *LGALS1*. (B) Pathway Commons analysis showing interactions between *SLC39A4* and *VAPA*, and between *GPR55*, *LGALS3*, and *LGALS1*.

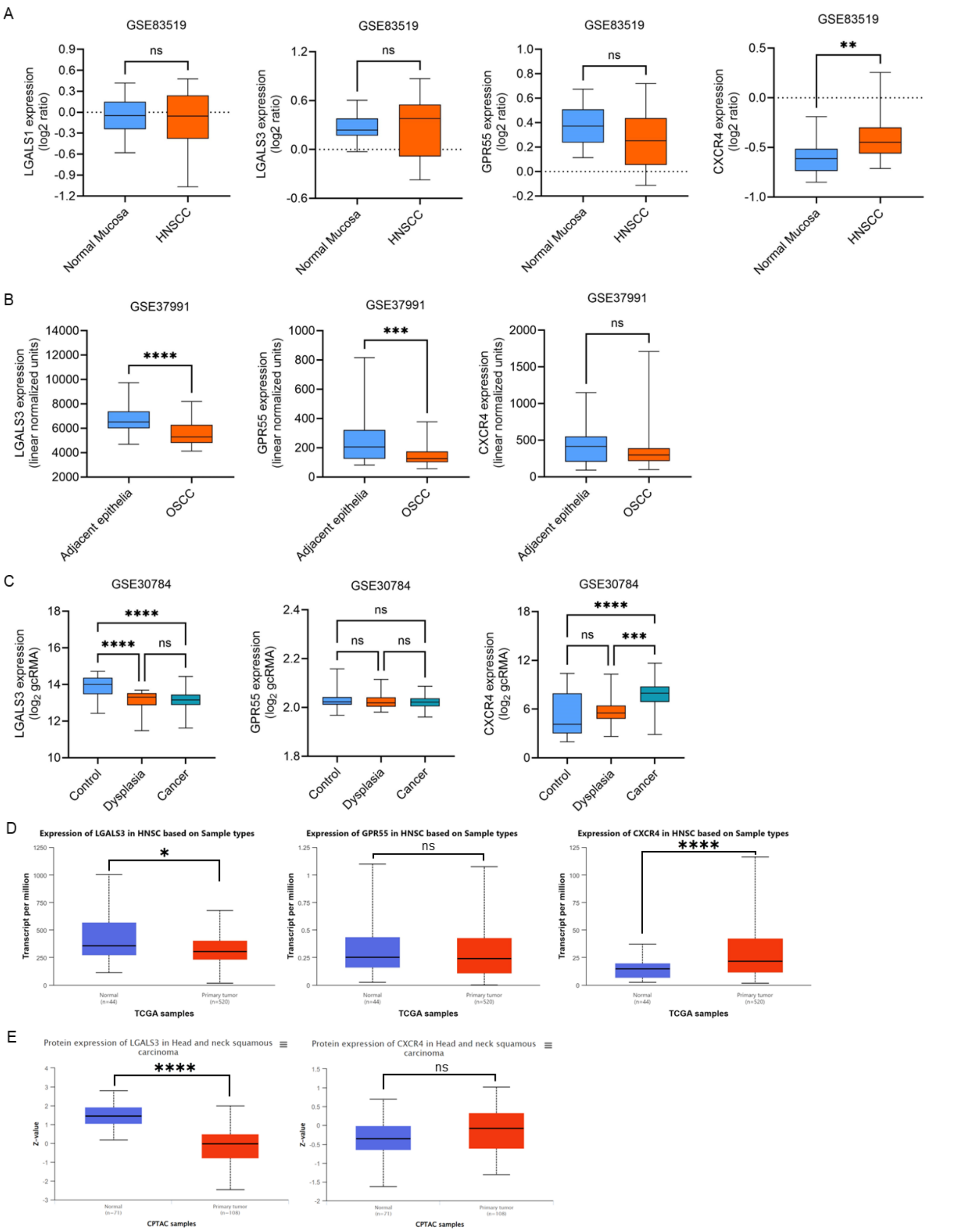

Supplementary Figure 4. Expression of *LGALS1*, *LGALS3*, *GPR55*, and *CXCR4* in HNSCC. (A–E) Expression profiles from GSE83519, GSE37991, GSE30784, TCGA, and CPTAC datasets.

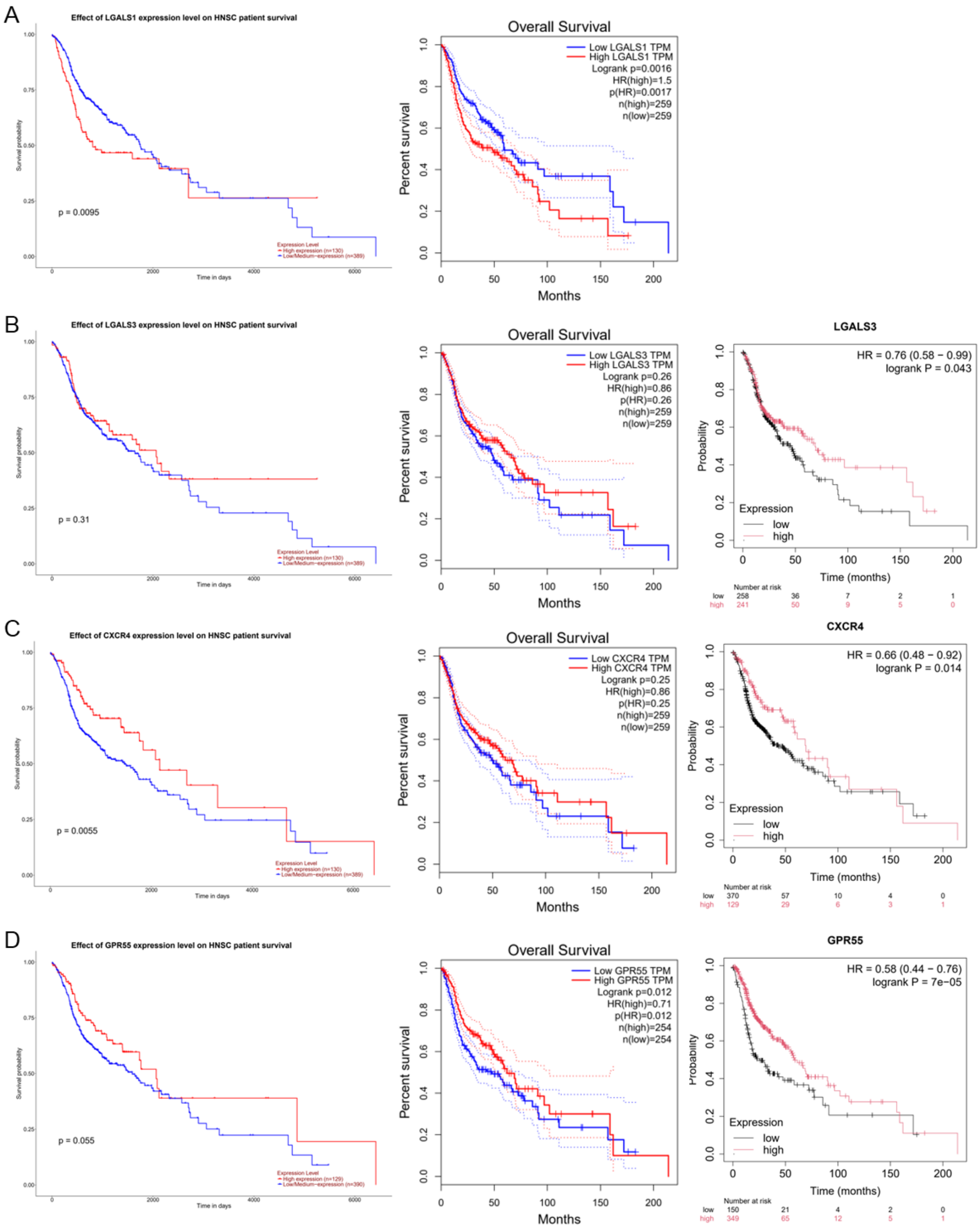

Supplementary Figure 5. Survival analysis of *LGALS1*, *LGALS3*, *GPR55*, and *CXCR4* in HNSCC. (A–D) Kaplan–Meier, UALCAN, and GEPIA survival curves showing associations between gene expression and survival.

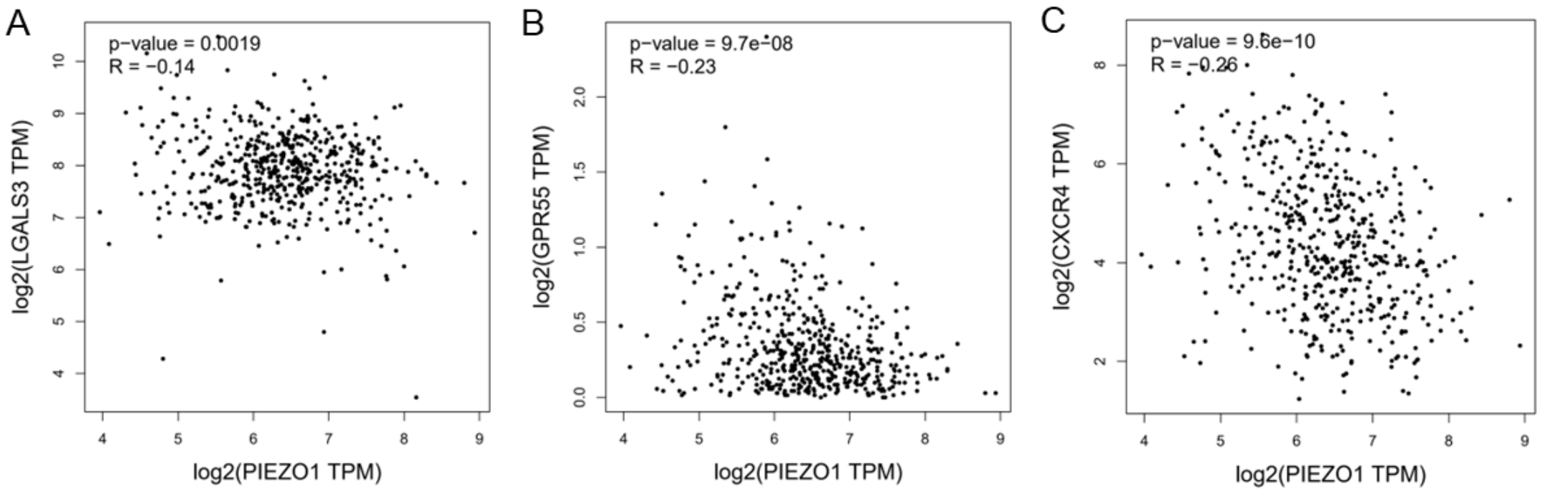

Supplementary Figure 6. Correlation of *PIEZO1* with *LGALS3*, *GPR55*, and *CXCR4* in HNSCC. (A–C) Scatterplots from GEPIA showing correlations between *PIEZO1* and *LGALS3*, *GPR55*, and *CXCR4*.
